# Juglanin inhibits lung cancer by regulation of apoptosis, ROS and autophagy induction

**DOI:** 10.18632/oncotarget.21317

**Published:** 2017-09-28

**Authors:** Liang Chen, Ya-Qiong Xiong, Jing Xu, Ji-Peng Wang, Zi-Li Meng, Yong-Qing Hong

**Affiliations:** ^1^ Department of Respiration, Huai’an First People’s Hospital, Nanjing Medical University, Huai’an, Jiangsu 223300, China

**Keywords:** juglanin, lung cancer, apoptosis, ROS, autophagy

## Abstract

Juglanin (Jug) is obtained from the crude extract of *Polygonum aviculare*, exerting suppressive activity against cancer cell progression *in vitro* and *in vivo*. Juglanin administration causes apoptosis and reactive oxygen species (ROS) in different types of cells through regulating various signaling pathways. In our study, the effects of juglanin on non-small cell lung cancer were investigated. A significant role of juglanin in suppressing lung cancer growth was observed. Juglanin promoted apoptosis in lung cancer cells through increasing Caspase-3 and poly ADP-ribose polymerase (PARP) cleavage, which is regulated by TNF-related apoptosis-inducing ligand/Death receptors (TRAIL/DRs) relied on p53 activation. Anti-apoptotic members Bcl-2 and Bcl-xl were reduced, and pro-apoptotic members Bax and Bad were enhanced in cells and animals receiving juglanin. Additionally, nuclear factor-κB (NF-κB), phosphoinositide 3-kinase/protein kinase B (PI3K/AKT) and mitogen-activated protein kinases (MAPKs) activation were inhibited by juglanin. Further, juglanin improved ROS and induced autophagy. ROS inhibitor *N-acetyl-l-cysteine* (NAC) reversed apoptosis induced by juglanin in cancer cells. The formation of autophagic vacoules and LC3/autophagy gene7 (ATG7)/Beclin1 (ATG6) over-expression were observed in juglanin-treated cells. Also, juglanin administration to mouse xenograft models inhibited lung cancer progression. Our study demonstrated that juglanin could be a promising candidate against human lung cancer progression.

## INTRODUCTION

Lung cancer is reported as a leading cause for cancer death worldwide, accompanied with ascending incidence and mortality [1, 2]. Of all lung cancer cases, the non-small cell lung cancer (NSCLC) is the most common type, accounting for approximately 85% [3]. Recently, some progress has been made in clinical and experimental oncology. But the prognosis for NSCLC is still limited with an overall 5-year survival rate of about 15% [4]. Thus, finding effective treatment and understanding the molecular mechanism of lung cancer progression are currently essential.

**Figure 1:**
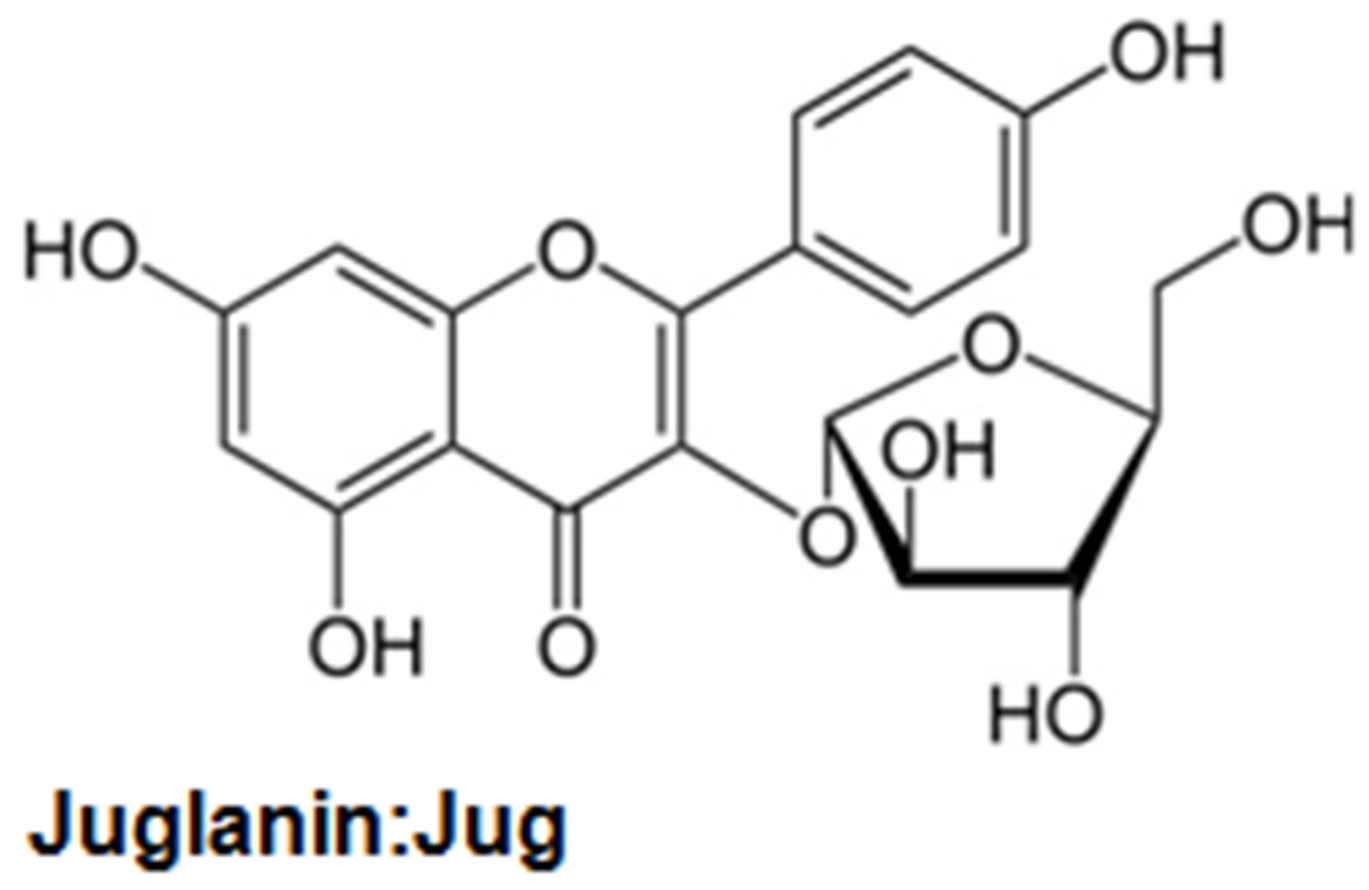
The chemical structure of juglanin

Presently, compound purified from the natural plants is widely explored for their biological activity, including anti-inflammation and anti-cancer [5, 6]. Further, compounds from plants are also of interest for their low cost, multiple targeting properties, relative absence of cytotoxicity and high availability [7, 8]. Currently, natural compounds are investigated in the development of different tumor calls *in vitro* and *in vivo* [9]. Juglanin (C_20_H_18_O_10_, Figure 1), also known as kaempferol 3-O-arabinofuranoside, is a kind of aldose reductase inhibitors [10, 11]. It is isolated from the crude *Polygonum aviculare*, which has a potential value for different biological activities, such as anti-inflammatory response and anti-tumor [12]. Juglanin has been explored in human breast cancer development and progression through apoptosis and autophagy, which was related to intracellular reactive oxygen species (ROS) accumulation [13]. Studies reported that juglanin could enhance autophagy and apoptosis in cancer cells *in vitro* and mouse xenograft models *in vivo* [14]. In addition, juglanin was reported to suppress NF-κB phosphorylation via p65 inactivity, exhibiting inhibitory effects on cellular senescence in human dermal fibroblasts [15]. However, the study of juglanin used in NSCLC is little to be reported, and there might be new molecular mechanisms or signaling pathways by which juglanin affects the development of lung cancer.

Apoptosis has been considered as cell death for tissue development and homeostasis in organisms [16–18]. The apoptotic cells are experienced with various molecular alterations via regulating different pro- and anti-apoptotic molecules [19]. The pro-apoptotic molecules include Bax, Bad, and Bak, while the latter involves Bcl-2, Bcl-xl and Mcl-1 [20, 21]. Caspases, including initiators Caspase-8 and Caspase-9, as well as effector Caspase-3, could be activated for the apoptotic members alteration [22]. Initiator Caspase-8 and Caspase-9 activate Caspase-3, cleaving PARP and inducing apoptosis eventually [23, 24]. Hence, apoptosis induction and potentiation has been regarded as tumor therapy [25]. According to previous studies, NF-κB is of great importance in activating anti-apoptotic members, including Bcl-2, Mcl-1, Bcl-xl as well as c-Flip, which inhibit apoptotic response [26]. Thus, suppressing NF-κB activation could be a notable therapeutic strategy to impede anti-apoptosis, and induce pro-apoptosis. IκBα has been well known in regulating NF-κB levels. IκBα and NF-κB form a complex, inhibiting NF-κB translocation into nuclear and suppressing anti-apoptotic members expression. In contrast, phosphorylated IκBα abolished IκBα/NF-κB complex, promoting NF-κB translocation into nuleus and causing anti-apoptotic response [27]. PI3K/AKT signaling pathway has been reported to inhibit apoptotic response through inducing p65 [28, 29].

Accumulating evidences have indicated that increased ROS generation is involved in cancer cells, which is induced by various drugs [30]. Increased ROS is responsible for cell death in various cancer cells [31]. Autophagy, as a cellular process, contains intracellular elements, which are engulfed, diggested as well as recycled through autophagosomes and autolyssosomes formation. Thus, it plays an essential role in cell survival under different conditions [32]. Cell death regulated by autophagy has been performed in tumor therapies [33–35].

We herein indicated that juglanin had anti-cancer effects on lung cancer *in vitro* and in a murine lung cancer-bearing mouse model via various methods. Mainly, juglanin induced apoptosis, ROS and autophagy in cancer cells. Of note, apoptosis triggered by juglanin was also influenced by ROS production. Additionally, we also found that for the first time, p53 promoted apoptotic cell death by activating a number of positive regulators of apoptosis. In contrast, suppression of p53 using its inhibitor dramatically reversed juglanin-induced cell death. Furthermore, NF-κB pathway, PI3K/AKT, and MAPKs (p38, ERK1/2 and JNK) pathways were all involved in juglanin-regulated lung cancer progression. Therefore, our study provides an effective candidate drug against human lung cancer development.

## RESULTS

### Juglanin induced cytotoxic effects and apoptosis in lung cancer cell lines

**Figure 2:**
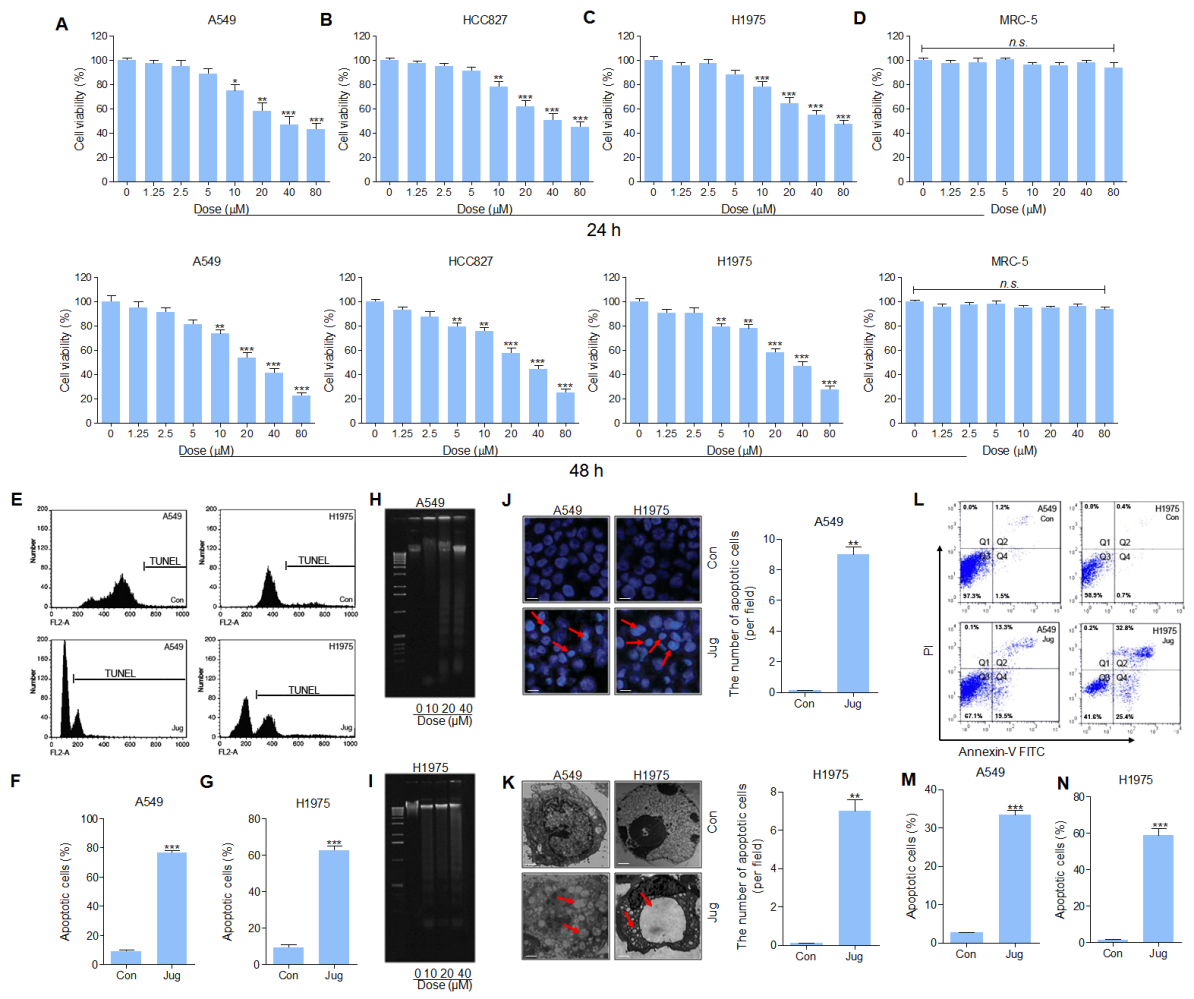
Juglanin induced cytotoxic effects and apoptosis in lung cancer cell lines Up, lung cancer cell lines of **(A)** A549, **(B)** HCC827, and **(C)** H1975 were administered with juglanin at different concentrations, ranging from 0 μM to 80 μM for 24 h. Then the cell viability was measured via MTT analysis. And the lung normal cell **(D)** MRC-5 was also treated with various concentrations of juglanin as indicated for 24 h. Then, MTT assays were conducted to calculate cell viability. Down, the lung cancer cell lines of (A) A549, (B) HCC827, and (C) H1975, and the lung normal cell of MRC-5 were treated with the presented concentrations of juglanin for 48 h, followed by MTT assays. The data are presented as mean ± S.E.M. of three independent experiments performed in duplicate. ^*^ P < 0.05, ^**^ P < 0.01 and ^***^ P < 0.001 compared to Control group without any treatment. **(E)** A549 and H1975 cells were cultured with 40 μM juglanin for 24 h. Then, flow cytometry assay was conducted to assess DNA contents in the sub-G1 phase. Con: the control group without any treatments. The representative images were exhibited. The quantification of the apoptotic **(F)** A549 and **(G)** H1975 cells in sub-G1 phase was calculated through statistic analysis. A549 and H1975 cells were treated with juglanin (10, 20 and 40 μM) for 24 h. The DNA fragmentation assay was conducted to determine DNA fragmentation in **(H)** A549 and **(I)** H1975 cells. **(J)** The representative images of apoptotic morphology of cancer cells treated with or without 40 μM juglanin for 24 h were shown. And the number of apoptotic cells per field was quantified. **(K)** Transmission electron microscopy was used to observe the chromatin condensation and the apoptotic cells after 40 μM juglanin treatment for 24 h. **(L)** The representative image of lung cancer cells experiencing apoptosis was displayed. The quantification of **(M)** A549 and **(N)** H1975 cells in apoptosis was assessed. The data are presented as mean ± S.E.M. of three independent experiments performed in duplicate. ^*^ P < 0.05, ^**^ P < 0.01 and ^***^ P < 0.001 compared to Control group (Con) without any treatment.

The cytotoxicity of juglanin in lung cancer cell lines, and normal cells of MRC-5, was assessed through MTT assay. The results indicated that the cell viability of A549, HCC827 and H1975 was reduced by juglanin treatment for 24 h. At the concentration of 5 μM or lower, no significant difference of the suppressed rate was observed, whereas from 10 μM, the cell viability was down-regulated in a dose-dependent manner (Figure 2A, 2B and 2C). While treated for 48 h at different concentrations, huge anti-proliferation property of juglanin on A549, HCC827 and H1975 was found (Figure 2A, 2B and 2C). On contrary, no cytotoxicity in MRC-5 cells was observed here (Figure 2D). The results above indicated that juglanin at the subtoxic concentration showed effective role in lung cancer cell lines proliferation without triggering toxicity in normal cells. According to the results above, 20, 30 and 40 μM juglanin was used for the following investigation.

Next, the number of sub-G1 DNA in A549 and H1975 cells pre-treated with juglanin was assessed. As shown in Figure 2E, 2F and 2G, pre-treatment of lung cancer cells with juglanin led to an increasing of cell during sub-G1 phase. From Figure 2H and 2I, DNA fragmentation in A549 and H1975 cells was apparently up-regulated for juglanin administration. In summary, the data suggested that apoptosis was involved in juglanin-induced cytotoxicity in lung cancer cells.

Apoptotic morphology character, including nuclear fragmentation and chromatin condensation, was determined in lung cancer cells treated with 40 μM juglanin (Figure 2J). Additionally, transmission electron microscopy showed that the cells without juglanin treatment, normal cell morphology was observed. However, after pre-treatment with juglanin, apoptotic bodies and chromatin condensation, features of cell apoptosis, were found (Figure 2K). Further, flow cytometry analysis suggested that juglanin administration dramatically resulted in an increasing of apoptotic rate in treated A549 and H1975 cells in comparison to the control ones (Figure 2L, 2M and 2N). The data above indicated that juglanin could induce cell death via promoting apoptosis in lung cancer cells.

### Juglanin promoted apoptosis in lung cancer cells through enhancing Caspases activation

**Figure 3:**
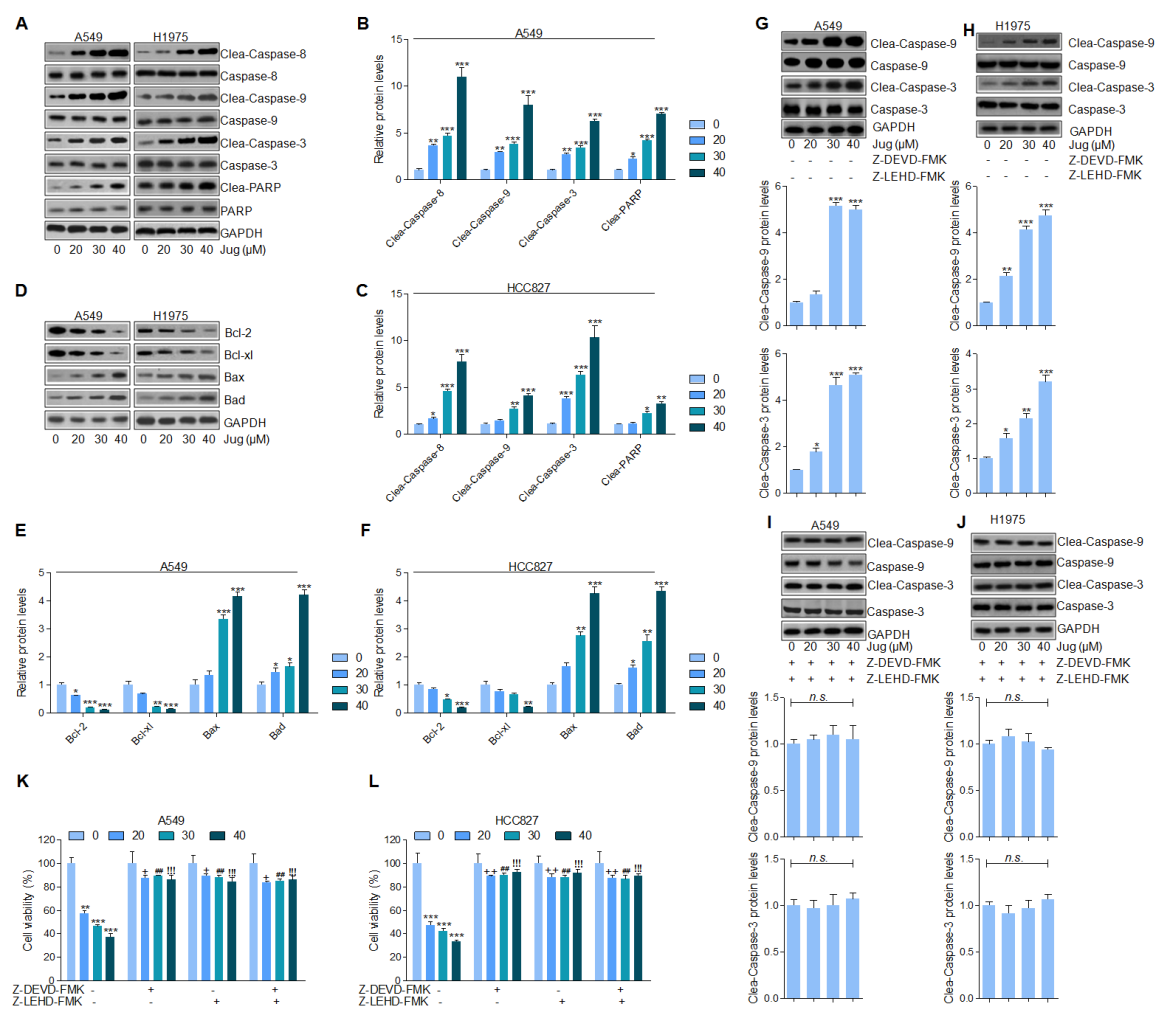
Juglanin promoted apoptosis in lung cancer cells through enhancing Caspases activation **(A)** A549 and H1975 cells were given juglanin at 0, 20, 30 and 40 μM for 24 h. Then, the cell protein was extracted and subjected to immunoblotting analysis for Caspase-8, Caspase-9, Caspase-3 and PARP determination. The quantification of western blot results of **(B)** A549 and **(C)** H1975 was shown. **(D)** Bcl-2 family, including Bcl-2, Bcl-xl, Bax and Bad, protein levels in A549 and H1975 cells after treatment under various conditions were evaluated by western blot analysis. The western blotting results in **(E)** A549 and **(F)** H1975 cells were quantified. **(G)** A549 and **(H)** H1975 lung cancer cells were treated with different concentrations of juglanin without Caspase-3 and Caspase-9 inhibitors of Z-DEVD-FMK and Z-LEHD-FMK for 24 h. Then cleaved Caspase-3 and Caspase-9 activity was calculated. **(I)** A549 and **(J)** H1975 cells were administered with the indicated concentrations of juglanin in the presence of Z-DEVD-FMK and Z-LEHD-FMK for 24 h, followed by determination of Caspase-3 and Caspase-9 cleavage. The cell viability of **(K)** A549 and **(L)** H1975 cells after Caspases inhibitors treatment was assessed by the use of MTT assay. The data are presented as mean ± S.E.M. of three independent experiments performed in duplicate. ^*^ P < 0.05, ^**^ P < 0.01 and ^***^ P < 0.001 compared to Control group (Con) without any treatment. ^++^ P < 0.01 and ^+++^ P < 0.001 compared to 20 μM juglanin treatment without Caspases inhibitors.^##^ P < 0.01 compared to 30 μM juglanin treatment without Caspases inhibitors.^!!!^ P < 0.001 compared to 40 μM juglanin treatment without Caspases inhibitors.

Caspase-8 activation causes the cleavage of Caspase-9 and Caspase-3 [17, 36]. As shown in Figure 3A, immunoblotting assays indicated that juglanin resulted in a significant cleavage of Caspase-8, Caspase-9 and Caspase-3. Furthermore, juglanin pre-treatment caused an obvious increasing of PARP in a dose-dependent manner in both A549 and H1975 cells (Figure 3B and 3C). Further, juglanin treatment apparently decreased Bcl-2 and Bcl-xl levels while accompanied with a prominent increase of Bax and Bad levels in A549 and H1975 cells (Figure 3D-3F).

In order to further confirm our supposing that Caspases was closely associated with juglanin-regulated apoptosis, Caspase-3 and Caspase-9 inhibitors, z-DEVD-FMK and z-LEHD-FMK respectively, were used. We found that Caspase-9 and Caspase-3 activity was noticeably up-regulated by juglanin treatment compared to the control group in cancer cells (Figure 3G and 3H). However, Caspases inhibitors z-DEVD-FMK and z-LEHD-FMK combinational treatment diminished Caspase-3 and Caspase-9 activation stimulated by juglanin in A549 and H1975 cells (Figure 3I and 3J). Additionally, the cell viability was dose-dependently down-regulated for juglanin treatment alone. Of note, the cell viability was also increased for Caspases inhibitors usage (Figure 3K and 3L). Taken together, the data above illustrated that Caspases-regulated apoptosis signaling pathway was a main molecular mechanism by which juglanin exhibited suppressive role in the gorwth of A549 and H1975 cells.

### Juglanin-promoted apoptosis in lung cancer cells was dependent on p53 signaling pathway

**Figure 4:**
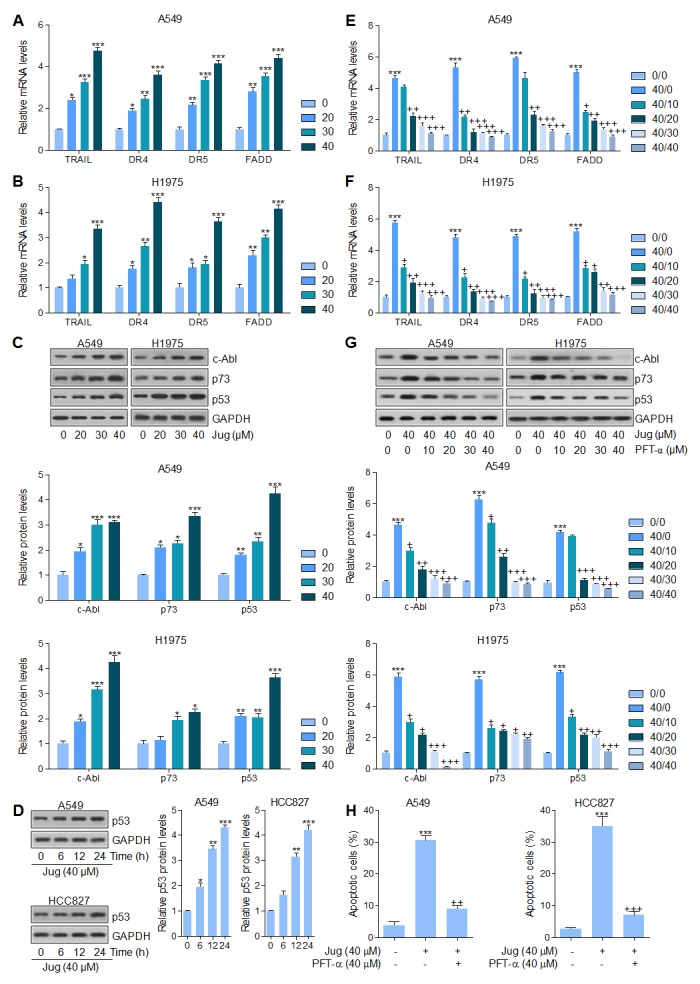
Juglanin-promoted apoptosis in lung cancer cells was dependent on p53 signaling pathway A549 and H1975 cells were exposed to various concentrations of juglanin for 24 h. RT-qPCR analysis was conducted to calculate the mRNA levels of TRAIL, DR4, DR5 and FADD in **(A)** A549 and **(B)** H1975 cells. **(C)** Up, representative images of c-Abl, p73 and p53 detected by western blot analysis were shown in both A549 and H1975 cells. Down, the quantification of c-Abl, p73 and p53 protein levels following the immunoblotting assays. **(D)** Left, A549 and H1975 cells were treated with 40 μM juglanin for the indicated time. Then, the p53 protein levels were calculated by the use of specific antibody. Right, the quantification of the immunoblotting data was shown. **(E)** A549 and **(F)** H1975 cells were treated with 40 μM juglanin in the presence or absence of different concentrations (0, 10, 20, 30 and 40 μM) of p53 inhibitor PFT-α for 24 h, followed by RT-qPCR analysis to detect TRAIL, DR4, DR5 and FADD mRNA levels. **(G)** Up, A549 and H1975 cells were treated with juglanin (40 μM) and various concentrations, ranged from 0 to 40 μM, of p53 inhibitor PFT-α for 24 h. Next, western blot assays were used to determine TRAIL, DR4, DR5 and FADD protein levels. Down, the quantification of immunoblotting data was exhibited. **(H)** A549 and H1975 cells were treated with 40 μM juglanin with or without PFT-α (40 μM) for 24 h ahead of flow cytometry analysis to assess apoptotic cells. The data are presented as mean ± S.E.M. of three independent experiments performed in duplicate. ^*^ P < 0.05, ^**^ P < 0.01 and ^***^ P < 0.001 compared to Control group (Con) without any treatment.

FADD is an important down-streaming signal of TRAIL pathway, regulated by p53, which could activate Caspase-8 and eventually cause apoptosis in cells [37, 38]. DR4 and DR5 were also explored for their role as TRAIL receptors, contributing to apoptosis [39]. As shown in Figure 4A and 4B, TRAIL, DR4, DR5 and FADD mRNA levels were highly expressed after juglanin treatment in both A549 and H1975 cells in a dose-dependent manner. Additionally, c-Abl/p73 signaling pathway could be activated for FADD/Caspase-8 activation, which has a positive relationship with Caspase-3 [37, 40]. Juglanin treatment caused an abundance protein expression of c-Abl, p73 and p53, which was comparable to the control ones (Figure 4C). Our study above has indicated that p53 could be up-regulated for juglanin treatment, which was in line with DR4 and DR5 alteration. Further, p53 expression was also showed in a time-dependent manner after 40 μM juglanin treatment for different time, ranging from 6 to 24 h in both lung cancer cells (Figure 4D). Thus, p53 might be a key point in regulating apoptosis under juglanin therapy. To further confirm p53 in juglanin-mediated apoptosis, p53 inhibitor PFT-α was used here. As shown in Figure 4E and 4F, PFT-α combined with juglanin dramatically reduced TRAIL, DR4, DR5 and FADD expression from gene levels. Of note, with the higher and higher PFT-α concentrations, the reduced levels of TRAIL, DR4, DR5 and FADD were lower and lower. Also, western blot analysis suggested that the up-regulated c-Able, p73 and p53 induced by juglanin were reversed by PFT-α in both A549 and H1975 cells (Figure 4G). Finally, in this regard, juglanin-induced high apoptosis proportion was apparently impeded for PFT-α pre-treatment (Figure 4H). The data above indicated that juglanin-triggered apoptosis was attributed to p53 pathway as well as its regulated TRAIL/DRs.

### Juglanin suppressed lung cancer progression through regulation of NF-κB, PI3K/AKT and MAPKs signaling pathways

**Figure 5:**
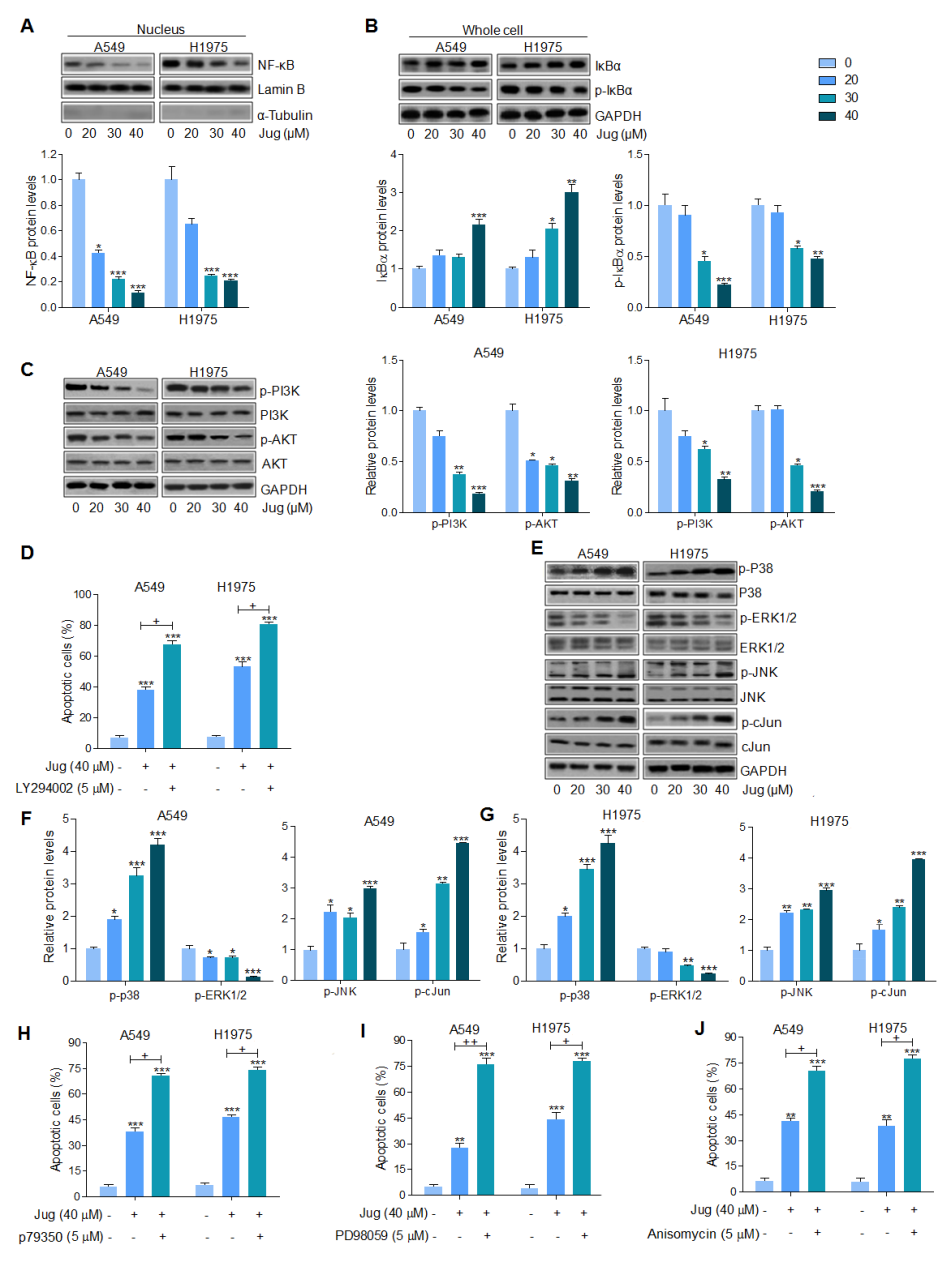
Juglanin suppressed lung cancer progression through regulation of NF-κB, PI3K/AKT and MAPKs signaling pathways **(A)** A549 and H1975 cells were treated with different concentrations of juglanin as indicated for 24 h. Then, the nuclues protein was extracted for western blot analysis of NF-κB by the specifc antibody. And Lamin B was applied as the loading control. **(B)** Total cell extracts were used to detect IκBα and phosphorylated IκBα protein levels. **(C)** A549 and H1975 cells were administered with different concentrations of juglanin for 24 h, followed by the whole cell protein extracts to calculate p-PI3K and p-AKT levels through western blot assays. **(D)** The flow cytometry analysis was used to determine apoptotic cells. A549 and HCC 827 cells were treated with 40 μM juglanin with or without PI3K/AKT inhibitor LY294002 (5 μM) for 24 h. **(E)** A549 and H1975 cells were treated with the indicated concentrations of juglanin for 24 h. And western blotting analysis was conducted to determine p-p38, p-ERK1/2 and p-JNK levels. The representative images were shown. The quantification of the immunoblotting results in **(F)** A549 and **(G)** H1975 cells. **(H)** A549 and HCC 827 cells were treated with 40 μM juglanin in the presence or absence of p38 activator p79350 (5 μM) for 24 h. The flow cytometry analysis was performed to detect apoptotic cells. **(I)** A549 and HCC 827 cells were exposed to 40 μM juglanin with or without ERK1/2 inhibitor 5 μM PD98059 for 24 h. The flow cytometry assay was used to determine apoptotic cells. **(J)** A549 and HCC 827 cells were treated by the use of 40 μM juglanin with or without JNK activator Anisomycin (5 μM) for 24 h. Apoptotic cells were determined by flow cytometry. The data are presented as mean ± S.E.M. of three separate experiments performed in duplicate. ^*^ P < 0.05, ^**^ P < 0.01 and ^***^ P < 0.001 compared to Control group (Con) without any treatment.^+^ P < 0.05 and ^++^ P < 0.01 were considered with significant difference.

Through western blot analysis, we found that nucleus NF-κB translocated from cytoplasm was inhibited by juglanin pre-treatment in a dose-dependent manner, which was in consistent with Bcl-2 and Bcl-xl changing in the presence of juglanin, suggesting that inhibition of NF-κB (p65) translocation suppressed anti-apoptotic members expression (Figure 5A). Also, in Supplementary Figure 1A and Figure 4B, A549 and H1975 cells cultured with juglanin expressed the low levels of p-NF-κB, Bcl-2 and Bcl-xl. In juglanin-absence cells, NF-κB inhibitor PDTC was used to suppress NF-κB activation. Of note, Bcl-2 and Bcl-xl were also inhibited for PDTC administration, similar to the effects of juglanin. In addition, Figure 5B showed that both in A549 and H1975 cells, IκBα was increased by juglanin, while p-IκBα was decreased. In our study, juglanin treatment down-regulated IκBα phosphorylation significantly, which might be important for juglanin-regulated apoptosis.

We further attempted to explore if PI3K/AKT signaling pathway was involved in juglanin-regulated apoptosis in lung cancer cells. From Figure 5C, p-PI3K and AKT were markedly down-regulated for juglanin pre-treatment in A549 and H1975 cells. In addition, flow cytometry analysis indicated that apoptosis induced by juglanin was higher compared to the control group, which was more intensive for PI3K/AKT inhibitor LY294002 combinational treatment (Figure 5D). Furthermore, LY294002 single treatment at different concentrations (1, 5 and 10 μM) up-regulated apoptotic cells in a dose-dependent manner, indicating that PI3K/AKT was indeed involved in lung cancer growth, which could be modulated for juglanin (Supplementary Figure 1C).

MAPKs, including p38, ERK1/2 and JNK, signaling pathway is essential for carcinogenesis [41]. And previous study suggested that juglanin inhibited breast cancer progression via regulating JNK expression [42]. As shown in Figure 5E, 5F and 5G, western blotting analysis suggested that p38 phosphorylation was up-regulated for juglanin treatment. And ERK1/2 phosphorylated levels were noticeably diminished for juglanin treatment in both A549 and H1975 cells. In contrast, JNK is reported to enhance apoptosis through various cellular stresses, such as DNA injury and oxidative stress, and crucial for cell proliferation as well as apoptosis induction [43]. c-Jun is a significant down-streaming signal of JNK, whose activation enhances c-Jun phosphorylation and promotes cell death [44, 45]. Our study indicated that juglanin treatment dose-dependently enhanced JNK and c-Jun phosphorylation. Blocking ERK1/2 activation by the use of ERK1/2 inhibitor, PD98059, facilitated apoptotic cells, which was stronger than juglanin alone treatment. Oppositely, juglanin combined with p38 and JNK activators, p79350 and Anisomycin, augmented the percentage of apoptosis, which was comparable to juglanin single therapy (Figure 5H, 5I and 5J). In Supplementary Figure 1D and 1E, the number of A549 and H1975 cells experiencing apoptosis was highly potentiated for p79350 and PD98059 pre-treatment. What’s more, JNK activator Anisomycin augmented the number of apoptotic lung cancer cells (Supplementary Figure 1F). In conclusion, the data above indicated that suppressing NF-κB, PI3K/AKT, and ERK1/2 phosphorylation, or enhancing p38 and JNK/c-Jun activity were responsible for the role of juglanin in inducing apoptosis.

### Juglanin enhanced ROS production in lung cancer cells

**Figure 6:**
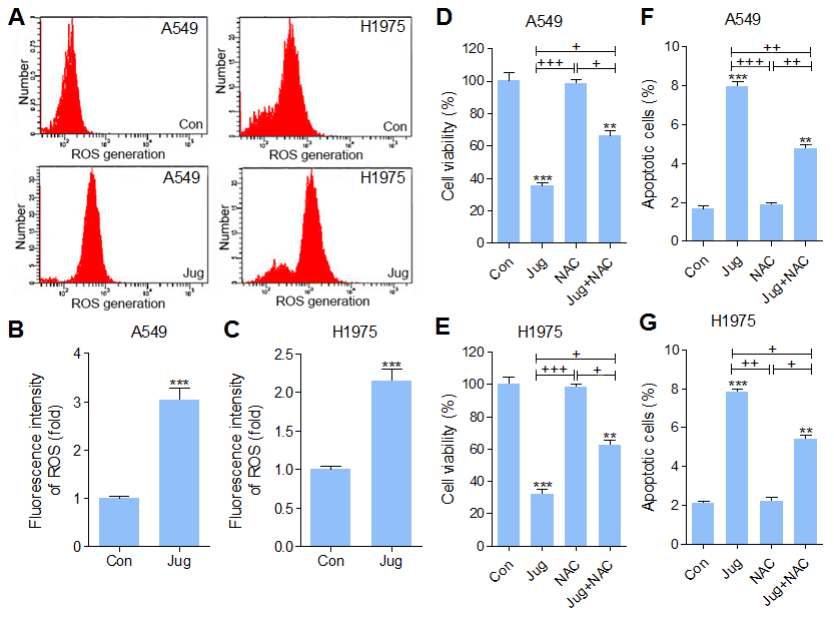
Juglanin enhanced ROS production in lung cancer cells **(A)** A549 and H1975 cells were pre-treated with 40 μM juglanin for 24 h. Then, the ROS generation was calculated. The representative images were shown here. The quantification of ROS production in **(B)** A549 and **(C)** H1975 cells was represented as relative fluorescent intensity. **(D)** A549 and **(E)** H1975 cells were treated with 10 mM NAC (ROS scavenger) for 2 h, and 40 μM juglanin for 24 h, or the combination of NAC for 2 h during exposure to juglanin for 24 h. Then, the cell viability was determined by MTT analysis. **(F)** A549 and **(G)** H1975 cells were administered with 10 mM NAC for 2 h, and 40 μM juglanin for 24 h, or NAC for 2 h during juglanin treatment for 24 h, followed by apoptotic cells determination through flow cytometry assay. The data are presented as mean ± S.E.M. of three separate experiments performed in duplicate. ^**^ P < 0.01 and ^***^ P < 0.001 compared to Control group (Con) without any treatment.^+^ P < 0.05, ^++^ P < 0.01 and ^+++^ P < 0.001 were considered with significant difference. *n.s.* referred to no significant difference.

In order to explore the involvement of oxidative stress in juglanin cytotoxicity, intracellular ROS levels were measured. As shown in Figure 6A, 6B and 6C, juglanin pre-treatment significantly up-regulated intracellular ROS production. To further confirm the role of ROS in juglanin-triggered cytotoxicity, A549 and H1975 cells were exposed to ROS scavenger N-acetylcysteine (NAC). The cells were treated with 40 μM juglanin in the absence or presence of 10 mM NAC. As shown in Figure 6D and 6E, NAC showed no significant cytotoxicity on A549 and H1975 cells, which was contrast to cytotoxicity action of juglanin. NAC exposure to juglanin-treated cells showed restored cell viability. In addition, apoptosis induced by juglanin was reversed for NAC treatment in both cells (Figure 6F and 6G). According to previous reports, ROS promotes tumor cell death from regulating AKT and MAPKs activity [46, 47]. As shown in Supplementary Figure 2A, 2B and 2C, PI3K/AKT activation was also inhibited by juglanin. Interestingly, cells cultured with NAC apparently up-regulated PI3K and AKT phosphorylation. Similarly, p38 and ERK1/2 were highly phosphorylated in NAC-treated cells. Of note, juglanin combined with NAC dramatically reduced p38 and ERK1/2 activation caused by NAC. In contrast, phosphorylated JNK was depressed for NAC. And in NAC-treated cells, advanced JNK phosphorylation was observed due to juglanin treatment (Supplementary Figure 2D, 2E and 2F).

### Juglanin induced lung cancer cell death through autophagy induction

**Figure 7:**
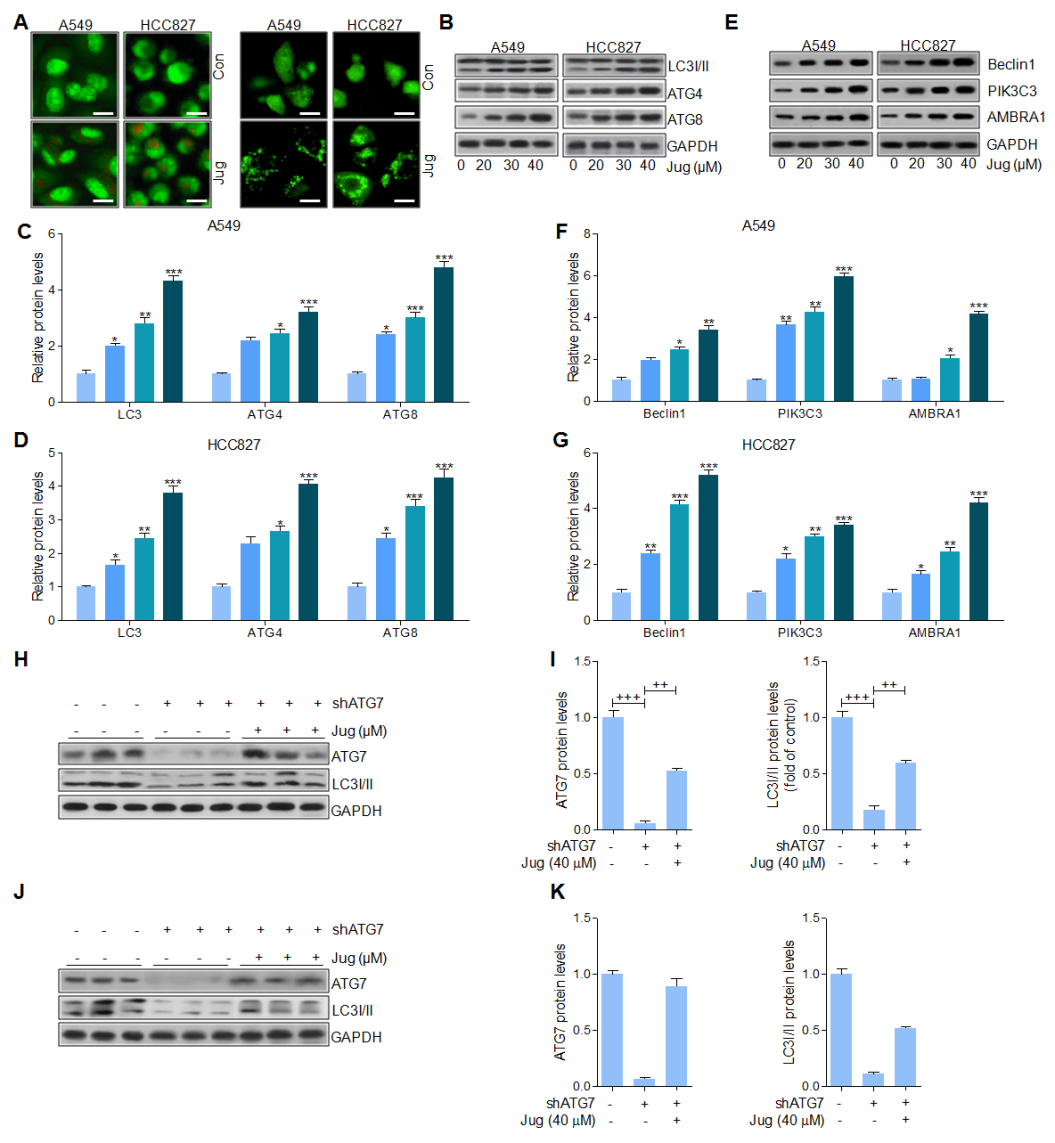
Juglanin induced lung cancer cell death through autophagy induction **(A)** A549 and H1975 cells were treated with 40 μM juglanin for 24 h. Next, the acidic vacuoles formation was analyzed by Acridine Orange (AO) staining (Left). And LC3-GFP puncta was used to analyze the autophagy condition of lung cancer cells treated as indicated (Right). **(B)** A549 and H1975 cells were administrated with various concentrations of jugalnin for 24 h. And western blot analysis was used to calculate LC3I/II, ATG7 and ATG3 protein levels. The quantification of the immunoblotting data from **(C)** A549 and **(D)** H1975 was shown. **(E)** The protein expression levels of autophagy induction-related signals, including Beclin1, and PIK3C3 were calculated through western blot analysis. And the quantification of these signals in **(F)** A549 and **(G)** H1975 cells was displayed. **(H)** ATG7 was knockdown in A549 cells treated with or without juglanin at 40 μM. Then, western blot analysis was used to evaluate ATG7 and LC3I/II levels. **(I)** The quantification of ATG7 and LC3 following immunoblotting in A549 cells was displayed. **(J)** ATG7 was silenced in H1975 cells treated with or without 40 μM juglanin. Then, western blot analysis was performed to determine ATG7 and LC3I/II levels. **(K)** The quantification of ATG7 and LC3 according to the immunoblotting in H1975 cells was shown. The data are presented as mean ± S.E.M. of three separate experiments performed in duplicate. ^**^ P < 0.01 and ^***^ P < 0.001 compared to Control group (Con) without any treatment.^+^ P < 0.05, ^++^ P < 0.01 and ^+++^ P < 0.001 were considered with significant difference.

Autophagy is reported to be involved in various tumors progression through accelerating cell death [31, 32, 48]. Autophagosomes, short-lived organelles, combine with acidic lysosomes to generate autolysosomes in which the sequestered content is degenerated under various conditions [49]. Acridine orange (AO) exhibited bright red color in acidic vesicles, while green fluorescence in nuclear and cytoplasm [50]. AO staining showed the exterior of acidic vesicular organelles in cells after juglanin pre-treatment (Figure 7A, left). We then transiently transfected A549 and H1975 cells with GFP-LC3 and incubated them with juglanin for 24 h. Our results indicated that juglanin significantly increased the percentage of cells containing GFP-LC3 puncta, whereas the Con group induced no or very weak autophagic effects (Figure 7A, right). LC3 expression has been well known in autophagy induction [51]. Data in this part indicated that juglanin caused LC3 transition in a concentration-dependent manner in cells (Figure 7B, 7C and 7D). Analogously, ATG7 and ATG3, two crucial genes in autophagy induction, were up-regulated due to juglanin treatment compared to the control group (Figure 7B, 7C and 7D). Further, Beclin1 is identified as tumor suppressor, which is proved to activate lipid kinase PIK3C3 to accumulate ATG, contributing to autophagy [52]. In our study, Beclin1 and PIK3C3 were accumulated in an abundance for juglanin treatment (Figure 7E, 7F and 7G). The data above indicated that autophagy induction via LC3 induction might be elicited by juglanin. To further confirm the role of ATG7 in autophagy formation, shRNA specific against ATG7 was included. In A549 cells, ATG7 was apparently down-regulated for shRNA treatment. Of note, juglanin dramatically recovered ATG7 protein expression. Further, ATG7 silence blocked LC3 expression, and juglanin administration apparently enhanced LC3 expression (Figure 7H and 7I). Further, in H1975 cells similar results were observed (Figure 7J and 7K). Together, the data above indicated that Beclin1 high expression, leading to LC3 activation, triggered by juglanin was also involved in suppressing lung cancer progression.

### Juglanin treatment suppressed tumor xenograft growth *in vivo* in a subcutaneous tumor model

**Figure 8:**
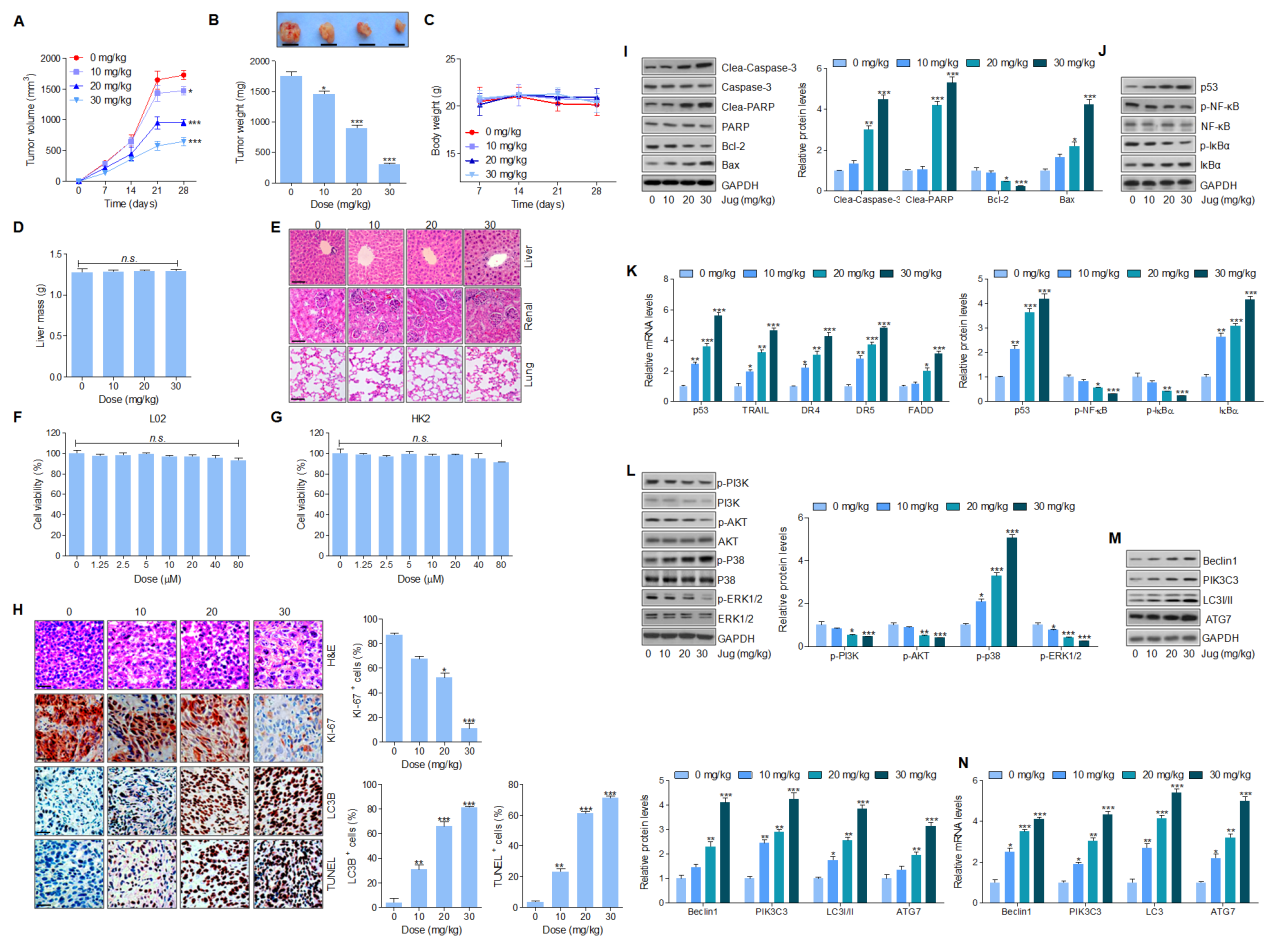
Juglanin treatment suppressed tumor xenograft growth *in vivo* in a subcutaneous tumor model The lung cancer cell A549 was subcutaneously injected into the dorsal flanks of 40 athymic nude mice. When the size of tumor reached to about 50 mm [3], the experimental animals were i.p. with 0, 10, 20 and 30 mg/kg juglanin each day for consecutive 4 weeks. **(A)** The tumor volume was measured every two days. **(B)** At the end of the *in vivo* study, the tumor from each group was weighed. **(C)** The body weight of the nude mice was analyzed. **(D)** At the end of the study, the liver from each mouse was obtained and weighed. **(E)** 24 athymic nude mice athymic nude mice were only treated with juglanin under different conditions (0, 10, 20 and 30 mg/kg) for consecutive 4 weeks. Finally, the representative images of liver, renal and lung sections analyzed by H&E staining were shown. **(F)** Liver normal cells L02 and **(G)** renal normal cells HK2 were treated with different concentrations (0,1.25, 2.5, 5, 10, 20, 40 and 80 μM) of juglanin for 24 h. Then, the cell viability was calculated through MTT assays. **(H)** The analysis of tumor tissue samples KI-67, autophagy (LC3B) and apoptosis (TUNEL) after treatment with juglanin under different conditions. The quota of tumor KI-67 positive cells, autophagy levels and apoptosis. The ratio of different positive cells was calculated from three fields with the highest density of positive-expressed cells in every section form each group. **(I)** Western blot analysis was used to calculate Caspase-3, PARP, Bcl-2 and Bax protein levels in the tumor tissue samples obtained from the mice under different conditions. The quantification of these proteins was shown. **(J)** p53, p-NF-κB and p-IκBα protein levels were calculated through western blotting analysis. **(K)** RT-qPCR assays were carried out to determine p53, TRAIL, DR4, DR5 and FADD mRNA levels in tumor tissue specimens. **(L)** PI3K, AKT, p38 and ERK1/2 phosphorylation was assessed by western blotting analysis. **(M)** Beclin1, PIK3C3, LC3I/II and ATG7 protein expression levels were measured via immunoblotting analysis. (J) The quantification of Beclin1, PIK3C3, LC3I/II and ATG7. **(N)** Beclin1, PIK3C3, LC3I/II and ATG7 gene levels were evaluated by RT-qPCR analysis. The results are presented as mean ± S.E.M. n=10. ^*^ P < 0.05, ^**^ P < 0.01 and ^***^ P < 0.001 compared to Control group (Con) without any treatment.

In this regard, the anti-cancer effect of juglanin was assessed in a xenograft tumor model through A549 cancer cells transplantation into the athymic nude mice. After 4 weeks juglanin treatment, the tumor volume and weight were considerably reduced in a concentration-dependent manner (Figure 8A and 8B). Additionally, no significant body weight and liver mass was observed, indicating an absence of toxicity in mice after juglanin treatment (Figure 8C and 8D). Furthermore, H&E staining was conducted to further evaluate the toxicity of juglanin in mice. The nude mice without any cancer cells in-plantation were received juglanin, and liver, renal as well as lung sections stained by H&E showed no significant difference (Figure 8E). Finally, normal cells from liver and renal were included to examine the effect of juglanin on cytotoxicity. Figure 8F and 8G revealed a lack of hepatic and renal cytotoxicity induced by juglanin ranging from 0 to 80 μM for 24 h. In summary, the findings here elucidated that juglanin exhibited anti-tumor function in lung cancer *in vivo*.

What’s more, immunohistochemical analysis indicated that KI-67 positive cells were highly reduced for jugalnin treatment in tumor sections (Figure 8H). LC3B quantification further evidenced that juglanin therapy suppressed A549 tumor growth via autophagy induction (Figure 8H). Also, in Figure 8H, TUNEL analysis illustrated that apoptosis was elicited by juglalnin to imped cancer growth *in vivo*. Moreover, western blotting analysis suggested that Caspase-3 and PARP cleavage were subcutaneously up-regulated, accompanied with down-regulated Bcl-2 and Bcl-xl protein levels as well as up-regulated Bax and Bad protein levels in mice receiving juglanin (Figure 8I). Additionally, p53 protein and mRNA levels were highly expressed in juglanin-treated mice, along with enhanced TRAIL, DR4, DR5 and FADD gene levels. Furthermore, p-NF-κB, p-IκBα, and IκBα showed identical trend with the findings in *in vitro* experiments, showed the descending p-NF-κB and p-IκBα, while the ascending IκBα (Figure 8J and 8K). Consistently, PI3K, AKT, and ERK1/2 phosphorylated protein levels were significantly suppressed for juglanin administration in A549 tumor samples, whereas p-p38 levels were highly induced by juglanin (Figure 8L). Furthermore, western blotting and RT-qPCR analysis revealed an abundance of LC3I/II, ATG7, Beclin1 and PIK3C3 in tumor samples from mice treated by juglanin (Figure 8M and 8N).

## DISCUSSION

Juglanin is a bioactive compound isolated from *Polygonum aviculare* and has been reported to possess suppressive role in breast cancer cells growth, as well as inhibitory activity on inflammation response in liver diseas [13]. However there are a limited number of studies referring to the *in vitro* and *in vivo* effects of juglanin on cancer progression, especially in the case of lung cancer. Lung cancer has been known as a leading cause for death in developed and developing countries. Among lung cancer patients, NSCLS accounts for about 85% of the cases, being the most common one [2, 53]. Though big progress has been made to treat lung cancer, just a small part of patients survived over a year after different therapies. Hence, new agent or novel compound is urgently needed to advance the traditional strategies for patients with lung cancer, especially NSCLC. In our study, we found that juglanin could induce apoptosis in tumor cells through activating Caspases, which was linked to p53/TRAIL signaling pathway. TRAIL is a member belonging to tumor necrosis factor family, which can trigger rapid apoptotic response of tumor cells without effects on normal cells. Thus, targeting apoptosis stimulation through TRAIL is widely used in clinical trials [54]. P53 signaling pathway was stimulated, along with high expression of DR4, DR5, FADD and p73. Detailed results indicated that juglanin reduced Bcl-2 and Bcl-xl, and enhanced Bax and Bad. Further, NF-κB, PI3K/AKT and ERK1/2 were suppressed for juglanin administration. ROS and autophagy were induced in jugalnin-treated groups, evidenced by the higher number of apoptotic cells and high expression of LC3, Beclin1and ATG7. Targeting these signaling pathways led to lung cancer cell death (Figure 8). Moreover, the role of juglanin in NSCLC cells was further validated in *in vivo* studies with xenografts nude mice where the natural compound inhibited lung cancer progression without secondary toxicity. Thus, juglanin could be of great potential value against human lung cancer in future.

In our study, MTT analysis found that juglanin suppressed the growth and proliferation of lung cancer cells. In comparison to the control group, juglanin administration induced significant apoptosis in tumor cells. Caspases activation is important for apoptotic response via p53/DRs/TRAIL and mitochondrial signaling pathways. The Caspases could be divided into two groups, the initiator Caspase-8 and Caspase-9, and the effector Caspase-3, based on their actions. Our data indicated that Caspase-8 and Caspase-9 were highly activated, and Caspase-3 was cleaved. Finally, cleaved PARP was up-regulated in juglanin-treated group and the nucleus condension was induced, leading to apoptosis and cell death in the end. The increase ratio of Bax/Bcl-2 is known as a key, resulting in apoptosis [52, 55]. Thus, promoting pro-apoptotic members can help to induce apoptosis, while anti-apoptotic molecules protect various cancer cells from apoptosis induction [21, 22, 25, 56]. Consistently, we found that juglanin up-regulated Bax and Bad expression, while Bcl-2 and Bcl-xl were down-regulated in cells and tumor samples.

P53, a typical tumor suppressor, suppresses cellular proliferation through regulating cell cycle arrest and apoptosis responding to various stresses, such as oncogene activation [57]. Previously, DR4 and DR5 can be activated by p53 [58]. TRAIL binding to DRs, stimulates FADD and Caspase-8, leading to apoptosis in cells [59, 60]. Our data indicated that juglanin apparently up-regulated p53, TRAIL, DR4, DR5 and FADD, which was in line with previous study [61]. PFT-α reversed p53 high expression induced by juglanin, and also the TRAIL, DR4, DR5 and FADD were reduced. The data obviously indicated that p53 played an essential role in apoptosis regulated by juglanin.

NF-κB signaling pathway is a key cellular signaling transduction pathway related to inflammation response, proliferation, and apoptosis [62]. NF-κB phosphorylation is an important event in various cancer cells progression, which is a potential target for cancer treatment [63]. In normal cells, NF-κB is maintained as an inactivated condition for its binding with IκBα. When IκBα is activated, IκBα phosphorylated form will be degraded from NF-κB. Then, NF-κB will be phosphorylated and then enter into the nuclear. Subsequently, the transcription of targeting genes is induced, enhancing tumor progression [62]. Additionally, NF-κB is reported to be a factor, activating many anti-apoptotic molecules expression, including Bcl-2 and Bcl-xl [64]. NF-κB suppression results in the down-regulation of anti-apoptotic proteins, leading to apoptosis and cell death [62–64]. In line with previous reports, NF-κB inhibition by juglanin or PDTC effectively reduced Bcl-2 and Bcl-xl, and enhanced Bax and Bad expression in cells. Hence, the NF-κB suppression by juglanin could be a crucial target for preventing lung cancer. The PI3K/AKT signaling pathway participates in various cellular processes, such as inflammation, proliferation, metabolism as well as apoptosis [65]. PI3K/AKT phosphorylation is known to accelerate cancer cells progression, which is considered as a target to investigate chemotherapies [66]. In our study, juglanin significantly inactivated PI3K/AKT expression. Of note, apoptosis was further induced by juglanin/LY294002 co-treatment. Therefore, on the one, PI3K/AKT signaling pathway was indeed involved in lung cancer development, and on the other its suppression might be an importantly promising target for juglanin against lung cancer progression *in vitro* and *in vivo*. Additionally, AKT is also reported to stimulate NF-κB phosphorylation [62]. Hence, AKT de-phosphorylation may result in NF-κB inactivation, subsequently down-regulating Bcl-2 and Bcl-xl expression and inducing apoptosis eventually.

p38 pathway-regulated apoptosis was also observed in different cell types [67]. Moreover, ERK1/2 activation is vital for carcinogenesis, and enhanced ERK1/2 is discovered in various human cancers [68]. Similarly, in our study, p38 was activated for juglanin treatment, which was consistent with previous study, indicating that p38 phosphorylation stimulate cancer cell death [67]. And its activator p79350 combined with juglanin considerably increased apoptotic cells. ERK1/2 were highly expressed in lung cancer cells, and juglanin significantly reduced ERK1/2 phosphorylation. Further, ERK1/2 inhibitor PD98059 apparently promoted the number of apoptotic cells after combination with juglanin. Hence, the augmentation of stress-response p38 and the inhibition of the ERK1/2 for survival might be involve din juglanin-induced apoptosis in lung cancer cells. Moreover, JNK is considered to enhance apoptosis through various cellular stresses, such as ROS and DNA damaging agent [69]. In addition, recent study has reported that JNK down-regulation is associated with apoptosis reduction [70]. Through c-Jun activation, JNK could stimulate pro-apoptotic genes, and down-regulate the anti-apoptotic molecules [71]. Our study showed that juglanin improved JNK and c-Jun activity, possibly accounted for apoptosis enhancement.

Intracellular ROS production is involved in cytotoxicity in many cancer cells [72]. Accordingly, accumulation of ROS could result in oxidative damage to cells [73]. Thus, ROS is relevant to cell death, which might be associated with apoptosis and autophagy through regulating various signaling pathways, including PI3K/AKT, NF-κB and MAPKs [74–76]. In our study, juglanin significantly caused an increase of ROS production in lung cancer cells. And NAC, ROS inhibitor, significantly reduced apoptosis levels in A549 and H1975 cells. In addition, NAC administration stimulated PI3K/AKT and ERK1/2 activation, and reduced p38 and JNK phosphorylation, which could be ameliorated by juglanin. Hence, increasing ROS generation was also a key in juglanin-treated lung cancer cells. Autophagy is well known as an important cause, leading to cell death [33–35]. As previously reported, the formation of LC3 bound autophagosomes is a character that represents autophagy formation [77]. Autophagy dis-regulation has been linked to various human diseases, such as cancers, which has been targeted as an alternative for cancer treatment [49, 50]. Beclin1, a tumor suppressor gene, could activate PIK3C3, which is important for ATG accumulation to start the formation of autophagosome [78]. Here, we reported that juglanin increased LC3, ATG7, Beclin1 and PIK3C3 expression, contributing to autophagy formation accompanied with the exterior of acidic vesicular organelles in lung cancer cells. Thus, just as previously reported, Beclin1 over-expression stimulates autophagy and decreases the carcinomagenesis in breast cancer cells [79]. The suppression of autophagy through ATG7 silence reduced LC3 expression, a key protein leading to autophagy formation, which could be reversed for juglanin treatment. The data indeed suggested that juglanin could induce cell death through promoting autophagy formation. *in vivo*, the tumor xenograft model indicated that juglanin efficiently suppressed tumor growth. The results also illustrated that the effective anti-cancer role of juglanin in lung cancer through inducing apoptosis, ROS and autophagy with little toxicity to animals.

According to previous study, juglanin could suppress breast cancer progression through inducing autophagy, apoptosis and ROS generation through promoting Caspases, LC3B and JNK expressions, seperately [42]. Consistently, juglanin could inhibit lung cancer development through the similar pathways. Notably, we found that juglanin could induce both intrinsic and extrinsic pathways to enhance apoptotic response in lung cancer cells, proved by the elevated Bad/Bax/Caspase-9 and TRAIL/FADD/Caspase-8, respectively. And the process might be attributed to p53 expressions. p53 mainly promotes apoptotic cell death by activating a number of positive regulators of apoptosis [57–61]. p53 suppression significantly down-regulated apoptosis in cancer cells with juglanin exposure. NF-κB, PI3K/AKT, and MAPKs pathways play an essential role in regulating tumor growth [62–65, 67–69]. Activation of the IKK/NF-kB signaling pathway leads to induction of target genes that can interfere with the apoptotic process. In the study, NF-κB, PI3K/AKT, and MAPKs (p38, ERK1/2 and JNK) pathways were all investigated for the first time fully revealing the underlying molecular mechanism by which juglanin prevented lung cancer progression. Both apoptosis and autophagy, belonged to programmed cell death (PCD), are important processes to induce cell death in any pathological format. Apoptosis invariably contributes to cell death, whereas autophagy can play either pro-survival or pro-death roles. Under some circumstances, apoptosis and autophagy can exert synergetic effects, whereas in other situations autophagy can be triggered only when apoptosis is suppressed [80, 81]. How apoptosis, autophagy and ROS pathways could be mapped and integrated with each other, what global properties are beginning to emerge from interactome network models, and how these properties may relate to lung cancer and its treatment have been investigated. But, due to their high levels of complexity, such networks have not been mapped completely. In summary, our study suggested that juglanin induced apoptosis and autophagy. We supposed that there might be a possible relationship between them to modulate cell death. As for this, further study in future is needed. Additionally, ROS could regulate apoptosis via various pathways mentioned above in our study, evidenced by reduced apoptotic proportion in NAC-treated cancer cells. However, if juglanin-induced ROS could influence autophagy, further study is still required in future.

**Figure 9:**
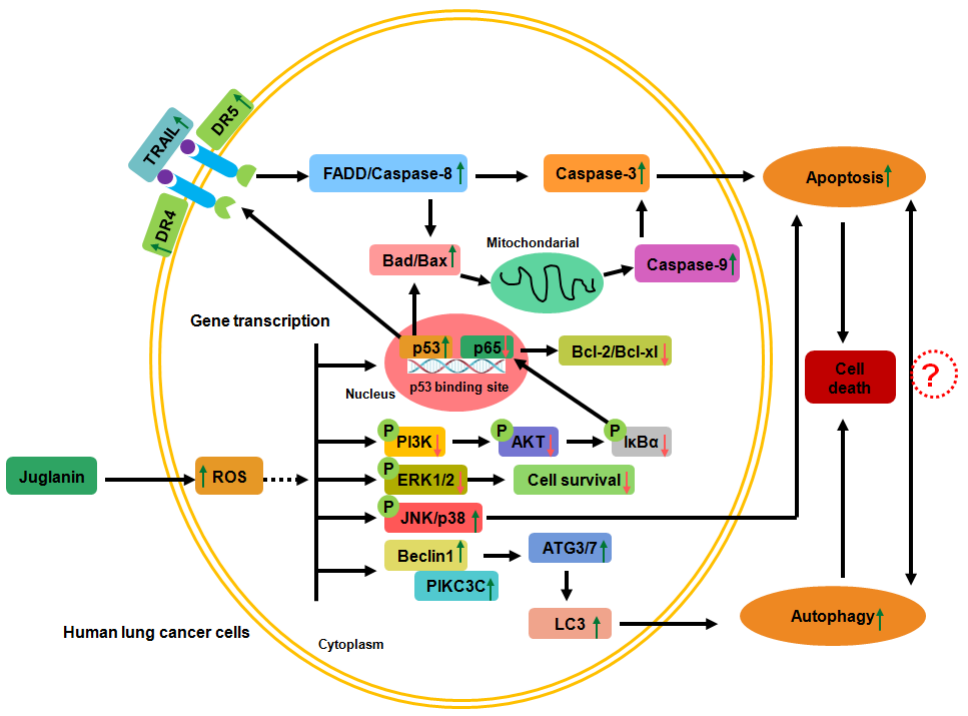
The working model regarding to the role of juglanin in lung cancer cells Juglanin improved ROS generation in human lung cancer cells. ROS stimulation down-regulated PI3K/AKT and ERK1/2 activation, while up-regulated JNK/P38 phosphorylation, leading to the cell survival reduction through suppressing p65 expression and promoting p53 levels. DR4 and DR5 were activated for p53 high expression, which bind to TRAIL and activate FADD/Caspase-8, resulting in Caspase-3 cleavage. On the other, p53 up-regulation accounts for Bad/Bax expression, augmenting Caspase-9 expression and activating Caspase-3, contributing to apoptosis eventually. Also, PI3K/AKT de-phosphorylation reduced NF-κB signaling pathway, causing anti-apoptotic molecules Bcl-2 and Bcl-xl expression suppression. Moreover, juglanin suppressed ERK1/2 activation and decreased cell survival, while JNK/P38 signaling pathways were activated, leading to apoptosis. Further, autophagy was also induced for juglanin treatment through Beclin1/PIK3C3 activation, triggering ATGs and LC3 activation. Finally, autophagy was elicited, causing lung cancer cell death. All of the signaling pathways were accounts for lung cancer cell death regulated by juglanin.

Taken together, our data supplied the evidence that juglanin triggered cell apoptosis, ROS, and autophagy via multiple targeting genes (Figure 9). Given the anti-cancer effects of juglanin without toxicity to normal cells and tissues, it could be considered as a promising therapeutic therapy to inhibit human lung cancer.

## MATERIALS AND METHODS

### Cells culture and reagents

The human non-small cell lung cancer cell line A549, HCC827 and H1975 were obtained from American Type Culture Collection (MD, USA). And human normal cell line of MRC-5, L02 and HK2 were purchased from the KeyGEN BioTECH (Nanjing, China). The human NSCLC cell lines A549, HCC827 and H1975 as well as L02 cells were cultured in RPMI 1640 (HyClone, UT, USA) containing 10% (v/v) fetal bovine serum (HyClone), and 1% penicillin/stretomycin (Invitrogen, CA, USA). MRC-5 and HK2 cells were grown in DMEM medium supplemented with (v/v) 10% heat-inactivated FBS and 1% penicillin/stretomycin (Invitrogen). Then, all cells were cultured at 37°C in a humidified atmosphere of 5% CO_2_. Juglanin (CAS: 5041-67-8, HPLC≥98%), isolated from leaves of English walnut (Juglans regia), was purchased from Shanghai ZeYe Biological Technology Co., LTD. (Shnghai, China). P53 inhibitor Pifithrin-α (PFT-α, Sigma-Aldrich, MO, USA), NF-κB inhibitor pyrollidine dithiocarbamate (PDTC, Sigma-Aldrich), PI3K inhibitor LY294002 (Selleck Chemicals, TEX, USA), p38 inhibitor p79350 (Beyotime, Jiangsu, China), ERK1/2 inhibitor PD98059 (Selleck Chemicals) and c-Jun N-terminal kinases (JNK) activator Anisomycin (Tocris Bioscience, Bristol, UK) were dissolved in dimethyl sulfoxide (DMSO, KeyGEN BioTECH) and freshly prepared for use.

### Cell viability analysis

3-(4,5-dimethylthiazol-2-thiazolyl)-2,5-diphenyl tetrazolium bromide (MTT) assay (KeyGENE BioTECH) was used to analyze the cell viability. Briefly, 1×10^4^ cells each well were cultured in 96-well plates (Corning, NY, USA). On the following day, different concentrations of juglanin (ranged from 0 to 80 μM) were administered to the medium for 24 h. Then the cell viability was calculated by MTT assay following the manufacturer’s instruction.

### Flow cytometry

To investigate the cell apoptosis, cancer cells were washed three times with ice-cold phosphate-buffered saline (PBS) and then re-suspended in binding buffer with 1×10^6^ cells/ml, followed by 20 μg/ml Annexin V-FITC (KeyGENE BioTECH) staining and 20 μg/ml propidium iodide (PI, Thermo Fisher Scientific, MA, USA) incubation for 15 min at the room temperature in dark based on the manufacturer’s instruction. Finally, 500 μl binding buffer was added into cells and flow cytometry (BD Bioscience, CA, USA) was used to assess the cells.

To assess the DNA degradation, the cells treated with juglanin were collected, washed with chilled PBS twice, and fixed using 75% ethonal at -20°C overnight. Then, cells were washed again with PBS, followed by incubation with 25 μg/ml RNase A (KeyGENE BioTECH) at 37°Cfor 30 min. Then, the cells were washed with PBS again and incubated using PI for 30 min at room temperature in dark. Cells were re-suspended in PBS and subjected to the flow cytometry (BD Bioscience). DNA degradation was calculated through assessing the cells in sub-G0/G1 peak.

### DNA fragmentation analysis

A549 and H1975 were treated with juglanin at different concentrations for 24 h. Then the cells were collected and washed with PBS, followed by re-suspension in lysis buffer (Beyotime, Shanghai, China) and incubation at 65°C overnight. Potassium acetate was then added to cells, which were incubated for 15 min at 4°C. Chloroform was added into the cells with vigorous mix. The cells were prepared for centrifugation at 12, 000 g for 10 min at room temperature. Then, the supernant was transferred into a new tube and ethanol was added into the tube for incubation overnight at -20°C. In the end, the sample was centrifugated at 12, 000 g for 10 min to obtain the DNA, which was washed, dried and dissolved in TE buffer (KeyGENE BioTECH). DNA was then assessed on 2% agarose gel.

### Nuclear condensation assay

A549 and H1975 cells were grown in RPMI 1640 medium, followed by juglanin treatment for 24 h. cells were then harvested by centrifugation. 4% formaldehyde was used to fix the pooled cell pellets. Then the cells were washed with the chilled PBS. DAPI (2.5 μg/ml) solution was used to stain cells at room temperature for 10 min, followed by washing, re-suspension and a fraction suspension was then smeared on a slide. Then, the slide was dried in air and mounted using VECTASHIELD mounting medium (Vector Laboratories, USA), which was observed under fluorescence microscope.

### Cell transfection

The short hairpin RNA against ATG7 was obtained from GENEray Biotech Co., Ltd (Shanghai, China). Then, Lipofectamine 2000 (Invitrogene, CA, USA) was used in the cells transfection experiments according to the manufacturer’s protocol.

### Assessment of ROS

The intracellular ROS production was calculated with the Reactive Oxygen Species Detection Assay Kit (Abcam, USA) for live-cell imaging (Invitrogen, USA) following the manufacturer’s instruction. 5×10^5^/ml lung cancer cells were plated in six-well plates and exposed to juglanin as indicated for 24 h. Cells were then stained with 10 μM dichlorofluorescin diacetate (DCFH-DA) in the dark room for 30 min at 37 °C. Next, the cells were washed with serum-free DMEM (2 ml) for three times, and the ROS generation was measured using a flow cytometry (BD Bioscience).

### Transmission electron microscopy analysis

The structural images of nuclear were analyzed by transmission electron microscopy. Cancer cells after juglanin treatment were harvested through centrifugation. Then, 2.5% glutaraldehyde (Nacalai, Japan) was used to fix the cells for 24 h, which was washed with phosphate buffer (PH 7.4) and post-fixed in osmium tetroxide purchased from Seebio Biotech, Inc. (Shanghai, China) in phosphate buffer. Then, the samples were dehydrated in the increasing doses of alcohol. Prophylene oxide was used to impregnated the samples, which were then embedded in the epoxy resin medium for embedding. Finally, the samples were stained using uranyl acetate (2%, w/v) and lead citrate (1%, w/v) and analyzed by a transmission electron microscope (JEM-1011, Japan).

### Acridine orange staining

Acridine orange (Cayman Chemical, MI, USA) was added into lung cancer cells at a final concentration 1 μg/ml for 15 min [82]. The representative images were obtained using a fluorescence microscope equipped with a digital camera (Olympus, Japan).

### Western blotting analysis

The cells were washed with chilled PBS and re-suspended in RIPA cell lysis buffer (Beyotime) completely for 30 min incubation on ice. And the tumor tissue samples were homogenated in RIPA cell lysis buffer completely. Then, they were centrifugated at 12, 000 g for 15 min at 4°C. The supernatant was then stored at -80°C for following research. The concentrations of protein were measured using bicinchonininc acid (BCA) kit (Thermo scientific). 30 μg/lane proteins were then separated on SDS-polyacrylamide gel electrophoresis, and transferred onto polyvinylidene fluoride (PVDF, Bio-Rad, CA, USA) membrane blocked with 5% slim milk in TBS buffer supplemented with 0.1% Tween-20 (Beyotime). Then the primary antibodies were used for incubation overnight at 4°C. Then secondary antibodies, including goat anti-mouse and goat anti-rabbit (Cell Signaling Technology, MA, USA), were used to horseradish peroxidase for 2 h at room temperature. The bound antibodies were observed through incubating membranes using chmilumiescence reagents (Thermo scientific) ahead of X-ray film (Eastman Kodak Company, NY, USA). The protein levels were quantified with Image J software (National Institutes of Health, Bethesda, MD). The primary antibodies used in our study were showed in Table 1.

**Table 1:** Primary antibodies for western blot analysis.

Primary antibodies	Dilution ratio	Corporation (CAS)
Rabbit anti- Caspase-3	1:1000	Abcam (ab2171)
Rabbit anti- Caspase-9	1:1000	Abcam (ab52298)
Rabbit anti- Caspase-8	1:1000	Abcam (ab32397)
Rabbit anti- PARP	1:1000	Abcam (ab32064)
Rabbit anti- Bcl-2	1:1000	Abcam (ab32124)
Rabbit anti-NF-κB	1:1000	Abcam (ab207297)
Rabbit anti- p- NF-κB	1:1000	Abcam (ab86299)
Rabbit anti- Bcl-xl	1:1000	Abcam (ab2568)
Rabbit anti- Bax	1:1000	Abcam (ab32503)
Rabbit anti-PI3K	1:1000	Abcam (ab86714)
Rabbit anti- p-AKT	1:1000	Abcam (ab38449)
Rabbit anti- AKT	1:1000	Abcam (ab8805)
Rabbit anti- Bad	1:1000	Abcam (ab32445)
Rabbit anti-cAbl	1:1000	Abcam (ab47315)
Mouse anti- p- PI3K	1:1000	Abcam (ab182651)
Rabbit anti- p73	1:1000	Abcam (ab40658)
Mouse anti-p53	1:1000	Cell Signaling Technology (ab2524)
Mouse anti- IκBα	1:1000	Cell Signaling Technology (ab4814)
Rabbit anti-p-IκBα	1:1000	Cell Signaling Technology (ab2859)
Rabbit anti-p38	1:1000	Cell Signaling Technology (ab8690)
Mouse anti- p-p38	1:1000	Cell Signaling Technology (ab4511)
Rabbit anti- ERK1/2	1:1000	Cell Signaling Technology (ab4695)
Rabbit anti-p-ERK1/2	1:1000	Cell Signaling Technology (ab9101)
Rabbit anti- JNK	1:1000	Cell Signaling Technology (ab9252)
Mouse anti-p-JNK	1:1000	Cell Signaling Technology (ab9255)
Rabbit anti-p-cJun	1:1000	Abcam (ab32385)
Mouse anti-cJun	1:1000	Abcam (ab32137)
Rabbit anti- LC3	1:1000	Abcam (ab48394)
Rabbit anti-ATG7	1:1000	Abcam (ab53255)
Rabbit anti-ATG3	1:1000	Abcam (ab108282)
Rabbit anti-Beclin1	1:1000	Abcam (ab62557)
Mouse anti-PIK3C3	1:1000	Abcam (ab5451)
Rabbit anti-Lamin B	1:1000	Abcam (ab133741)
Mouse anti-GAPDH	1:1000	Abcam (ab8245)

### Qauntitative real-time (RT)-qPCR analysis

Total RNA in cells and tumor tissue samples were extracted with TRIzol reagent (KeyGENE BioTECH) according to the manufaturer’s protocol, which was utilized to prepare cDNA with Thermo RT Kit cDNA (Thermo, USA). The quantitative real-time PCR was conducted by the use of TransStart Green qPCR SuperMix (TransGen Biotech, Beijing, China) with the Applied Biosystems 7500 Sequence Detection system. The primary sequences used in the present study were displayed in Table 2. Reactions were conducted following the protocol: pre-incubation for 10 min at 50°C, 5 min at 95°C, followed by 40 PCR cycles for 10 s at 95°C and 30 s at 60°C. GAPDH was used as the loading control. Fold induction values were evaluated using the 2^-ΔΔCq^ method, in which ΔCq means the differences in cycle threshold number between the target gene and GAPDH, and ΔΔCq represents the relative alteration in differences between the control and treatment groups under different conditions.

**Table 2:** Primer sequences of RT-PCR analysis.

Gene	Forward primers (5’-3’)	Reverse primers (5’-3’)
GAPDH	CATATCAGCAGACGAGGAC	ACTCAGCTACAAGACCACTACC
TRAIL	AGACACAAGAGTGAGTGCC	CGTCAGTCAGTGTATGTG
DR4	AAGGACATGCAGGCGATAC	TCGCATGCTAAGTTGGTGTGG
DR5	GCAGCAGTGAGTGGGCAGT	CCGCTGTACTGCTCTTCGGTT
FADD	AGGCAGCTGCGTGTAGCAG	TTACCTGCGCTTAATGCTGC
Beclin1	CAGGAAGACAGAGTAGTTC	CCGCGTAGTGTGGATGATG
PIK3C3	TCACCTTGATCTGACCCTGC	G TCCTCGTCTCATGTCTGCG
LC3	CTGACTGGTCCTCTCTATTCA	CTTATGGCGACAAATAGCCT
ATG7	GCAAGACAAGATTCGATACT	GCCAGACTACATGGAAATCTA

### GFP-LC3 puncta assessment

Cancer cells after various treatments were fixed with 4% paraformaldehyde and then mounted onto microscope slides with FluorSave™ Reagent (Calbiochem, USA). Localisation of GFP-LC3 was evaluated with a microscope (Nikon, Japan). Images were captured using a CCD digital camera Spot RT3™ under the Nikon ECLIPSE 80i microscope (Diagnostic Instruments, Inc., USA).

### Caspases activity assay

Cancer Cells after various treatments were collected and washed twice with cold PBS. Then, the cell pellets were lysed with lysis buffer (100 ml) (Beyotime). And Ac-DEVD-pNA and Ac-LEHD-pNA (substrate for Caspase-3 and Caspase-9, respectively) were used to incubate the lysates. After incubation at 37°C for 2 h, released p-nitroanilide was measured at 405 nm with a micro-plate reader. Caspase-3 and -9 activity was normalized to the protein content and expressed as a fold of that for the control cells.

### Athymic nude model experiment

6-week old Athymic nude mice were obtained from Nanjing Medical University Animal Center (Nanjing, China) and fed in a temperature (25 ± 2°C) and humidity-controlled environment (50 ± 10% humidity) with a standard 12 h:12 h light:dark cycle with the food and water in cages under germ-free conditions. All processes were in line with the Institutional Animal Care and Use Committee of Huai’an First People’s Hospital, Nanjing Medical University. Briefly, 5 × 10^5^ A549 cells were subcutaneously injected into the dorsal flanks of the nude mice. Tumor volume was estimated by assessing the two maximum perpendicular tumor diameters every three days. All tumor-bearing nude mice were randomly divided into 4 groups: (1) The Control (i.p. injection of normal saline); (2) Juglanin (10 mg/kg); (3) Juglanin (20 mg/kg); (4) Juglanin (30 mg/kg) every day for 28 days [12, 13]. Finally, the tumors were harvested and weighed. In addition, the body weight and liver weight were measured to calculate the toxicity of various treatments on mice *in vivo*. In parallel, animals without tumor bearing were treated with juglanin as mentioned above. Then, the liver, renal and lung tissues were obtained for haematoxylin and eosin (H&E) staining.

### Immunohistochemical analysis

The liver, renal and lung tissue samples were fixed with 4% formaldehyde, embedded in paraffin and then sectioned for H&E staining following the standard histochemical procedures [83]. The percentage of apoptotic cells in lung tumor sections were evaluated through TUNEL technique according to the manufacturer’s introduction (KeyGENE BioTECH), and were conducted to IHC staining for the measurement of KI-67and LC3B expression with the specific antibodies [84].

### Statistical analysis

Data were represented as mean ± S.E.M. Statistical analyses were carried out by the use of GraphPad PRISM (version 6.0; Graph Pad Software, USA) by ANOVA with the Dunnet’s least significant difference *post-hoc* tests. In all comparisons, a p-value < 0.05 will be considered as statistically significant.

## SUPPLEMENTARY MATERIALS FIGURES



## References

[1] Siegel RL, Miller KD, Jemal A (2016). Cancer statistics, 2016. CA Cancer J Clin.

[2] Chang A (2011). Chemotherapy, chemoresistance and the changing treatment landscape for NSCLC. Lung Cancer.

[3] Ettinger DS, Wood DE, Akerley W, Bazhenova LA, Borghaei H, Camidge DR, Cheney RT, Chirieac LR, D’Amico TA, Demmy TL, Dilling TJ, Dobelbower MC, Govindan R (2015). Non–small cell lung cancer, version 6.2015. J Natl Compr Canc Netw.

[4] Ying L, Zhu Z, Xu Z, He T, Li E, Guo Z, Liu F, Jiang C, Wang Q (2015). Cancer associated fibroblast-derived hepatocyte growth factor inhibits the paclitaxel-induced apoptosis of lung cancer A549 cells by up-regulating the PI3K/Akt and GRP78 signaling on a microfluidic platform. PLoS One.

[5] Wahlang B, Pawar YB, Bansal AK (2011). Identification of permeability-related hurdles in oral delivery of curcumin using the Caco-2 cell model. Eur J Pharm Biopharm.

[6] Adhami VM, Syed DN, Khan N, Mukhtar H (2012). Dietary flavonoid fisetin: a novel dual inhibitor of PI3K/Akt and mTOR for prostate cancer management. Biochem Pharmacol.

[7] Singh PK, Kotia V, Ghosh D, Mohite GM, Kumar A, Maji SK (2013). Curcumin modulates α-Syn aggregation and toxicity. ACS Chem Neurosci.

[8] Wang J, Sun G, Yu L, Wu F, Guo X (2013). Enhancement of the selective enzymatic biotransformation of rutin to isoquercitrin using an ionic liquid as a co-solvent. Bioresour Technol.

[9] Ren M, Deng X, Ai F, Yuan G, Song H (2015). Effect of quercetin on the proliferation of the human ovarian cancer cell line SKOV-3 *in vitro*. Exp Ther Med.

[10] Tchinda AT, Agbor G, Tsala DE, Yaya AJ, Nga EN, Talla E, Wauters JN, Federich M (2014). Antioxidant activity of flavonoids isolated from the fruits of Xylopia parviflora (A. Rich.) Benth. Int J Pharma Sci Drug Res.

[11] Poonam V, Raunak, Kumar G, Reddy LC, Jain R, Sharma SK, Prasad AK, Parmar VS (2011). Chemical constituents of the genus Prunus and their medicinal properties. Curr Med Chem.

[12] Kim HH, Oh MH, Park KJ, Heo JH, Lee MW (2014). Anti-inflammatory activity of sulfate-containing phenolic compounds isolated from the leaves of Myrica rubra. Fitoterapia.

[13] Zhou GY, Yi YX, Jin LX, Lin W, Fang PP, Lin XZ, Zheng Y, Pan CW (2016). The protective effect of juglanin on fructose-induced hepatitis by inhibiting inflammation and apoptosis through TLR4 and JAK2/STAT3 signaling pathways in fructose-fed rats. Biomed Pharmacother.

[14] Sun ZL, Dong JL, Wu J (2017). Juglanin induces apoptosis and autophagy in human breast cancer progression via ROS/JNK promotion. Biomed Pharmacother.

[15] Yang HH, Hwangbo K, Zheng MS, Son JK, Kim HY, Baek SH, Choi HC, Park SY, Kim JR (2014). Inhibitory effects of juglanin on cellular senescence in human dermal fibroblasts. J Nat Med.

[16] Suzanne M, Steller H (2013). Shaping organisms with apoptosis. Cell Death Differ.

[17] Riedl SJ, Salvesen GS (2007). The apoptosome: signalling platform of cell death. Nat Rev Mol Cell Biol.

[18] Canbay A, Taimr P, Torok N, Higuchi H, Friedman S, Gores GJ (2003). Apoptotic body engulfment by a human stellate cell line is profibrogenic. Lab Invest.

[19] Kuo Y, Lucero L, Michaels J, DeLuca D, Lukas RJ (2002). Differential expression of nicotinic acetylcholine receptor subunits in fetal and neonatal mouse thymus. J Neuroimmunol.

[20] Kamsani YS, Rajikin MH, Mohamed Nor Khan NA, Abdul Satar N, Chatterjee A (2013). Nicotine-induced cessation of embryonic development is reversed by gamma-tocotrienol in mice. Med Sci Monit Basic Res.

[21] Chen G, Cheng X, Zhao M, Lin S, Lu J, Kang J, Yu X (2015). RIP1-dependent Bid cleavage mediates TNFalpha-induced but Caspase-3-independent cell death in L929 fibroblastoma cells. Apoptosis.

[22] Cooley-Andrade O, Cheung K, Chew AN, Connor DE, Parsi K (2016). Detergent sclerosants at sub-lytic concentrations induce endothelial cell apoptosis through a caspase dependent pathway. Apoptosis.

[23] Sun H, Yang S, Li J, Zhang Y, Gao D, Zhao S (2016). Caspase-independent cell death mediated by apoptosis-inducing factor (AIF) nuclear translocation is involved in ionizing radiation induced HepG2 cell death. Biochem Biophys Res Commun.

[24] Skorko-Glonek J, Zurawa-Janicka D, Koper T, Jarzab M, Figaj D, Glaza P, Lipinska B (2013). HtrA protease family as therapeutic targets. Curr Pharm Design.

[25] de Bruin EC, Medema JP (2008). Apoptosis and non-apoptotic deaths in cancer development and treatment response. Cancer Treat Rev.

[26] Li-Weber M (2013). Targeting apoptosis pathways in cancer by Chinese medicine. Cancer Lett.

[27] Korashy HM, El-Kadi AO (2008). The role of redox-sensitive transcription factors NF-kappaB and AP-1 in the modulation of the Cyp1a1 gene by mercury, lead, and copper. Free Radic Biol Med.

[28] Romashkova JA, Makarov SS (1999). NF-kappaB is a target of AKT in anti-apoptotic PDGF signalling. Nature.

[29] Ozes ON, Mayo LD, Gustin JA, Pfeffer SR, Pfeffer LM, Donner DB (1999). NF-kappaB activation by tumour necrosis factor requires the Akt serine-threonine kinase. Nature.

[30] Zhao J, Zhang L, Li J, Wu T, Wang M, Xu G, Zhang F, Liu L, Yang J, Sun S (2015). A novel pyrazolone-based derivative induces apoptosis in human esophageal cells via reactive oxygen species (ROS) generation and caspase-dependent mitochondria-mediated pathway. Chem Biol Interact.

[31] Menzies FM, Fleming A, Rubinsztein DC (2015). Compromised autophagy and neurodegenerative diseases. Nat Rev Neurosci.

[32] Rubinsztein DC, Codogno P, Levine B (2012). Autophagy modulation as a potential therapeutic target for diverse diseases. Nat Rev Drug Discov.

[33] Sarkar S, Korolchuk VI, Renna M, Imarisio S, Fleming A, Williams A, Garcia-Arencibia M, Rose C, Luo S, Underwood BR, Kroemer G, O'Kane CJ, Rubinsztein DC (2011). Complex inhibitory effects of nitric oxide on autophagy. Mol Cell.

[34] Renna M, Schaffner C, Winslow AR, Menzies FM, Peden AA, Floto RA, Rubinsztein DC (2011). Autophagic substrate clearance requires activity of the syntaxin-5 SNARE complex. J Cell Sci.

[35] Mauvezin C, Nagy P, Juhasz G, Neufeld TP (2015). Autophagosome-lysosome fusion is independent of V-ATPase-mediated acidification. Nat Commun.

[36] Mocarski ES, Upton JW, Kaiser WJ (2012). Viral infection and the evolution of caspase 8-regulated apoptotic and necrotic death pathways. Nat Rev Immunol.

[37] Micheau O, Solary E, Hammann A, Dimanche-Boitrel MT (1999). Fas ligand–independent, FADD-mediated activation of the Fas death pathway by anticancer drugs. J Biol Chem.

[38] Scott FL, Stec B, Pop C, Dobaczewska MK, Lee JJ, Monosov E, Robinson H, Salvesen GS, Schwarzenbacher R, Riedl SJ (2009). The Fas-FADD death domain complex structure unravels signalling by receptor clustering. Nature.

[39] Sprick MR, Rieser E, Stahl H, Grosse-Wilde A, Weigand MA, Walczak H (2002). Caspase-10 is recruited to and activated at the native TRAIL and CD95 death-inducing signalling complexes in a FADD-dependent manner but can not functionally substitute caspase-8. EMBO J.

[40] Chakraborty J, Banerjee S, Ray P, Hossain DM, Bhattacharyya S, Adhikary A, Chattopadhyay S, Das T, Sa G (2010). Gain of cellular adaptation due to prolonged p53 impairment leads to functional switchover from p53 to p73 during DNA damage in acute myeloid leukemia cells. J Biol Chem.

[41] de la Cruz-Morcillo MA, Valero ML, Callejas-Valera JL, Arias-González L, Melgar-Rojas P, Galán-Moya EM, García-Gil E, García-Cano J, Sánchez-Prieto R (2012). P38MAPK is a major determinant of the balance between apoptosis and autophagy triggered by 5-fluorouracil: implication in resistance. Oncogene.

[42] Sui X, Kong N, Ye L, Han W, Zhou J, Zhang Q, He C, Pan H (2014). p38 and JNK MAPK pathways control the balance of apoptosis and autophagy in response to chemotherapeutic agents. Cancer Lett.

[43] El-Najjar N, Chatila M, Moukadem H, Vuorela H, Ocker M, Gandesiri M, Schneider-Stock R, Gali-Muhtasib H (2010). Reactive oxygen species mediate thymoquinone-induced apoptosis and activate ERK and JNK signaling. Apoptosis.

[44] Tournier C, Hess P, Yang DD, Xu J, Turner TK, Nimnual A, Bar-Sagi D, Jones SN, Flavell RA, Davis RJ (2000). Requirement of JNK for stress-induced activation of the cytochrome c-mediated death pathway. Science.

[45] Park KR, Nam D, Yun HM, Lee SG, Jang HJ, Sethi G, Cho SK, Ahn KS (2011). β-Caryophyllene oxide inhibits growth and induces apoptosis through the suppression of PI3K/AKT/mTOR/S6K1 pathways and ROS-mediated MAPKs activation. Cancer Lett.

[46] McCubrey JA, Lahair MM, Franklin RA (2006). Reactive oxygen species-induced activation of the MAP kinase signaling pathways. Antioxid Redox Signal.

[47] Chetram MA, Bethea DA, Odero-Marah VA, Don-Salu-Hewage AS, Jones KJ, Hinton CV (2013). ROS-mediated activation of AKT induces apoptosis via pVHL in prostate cancer cells. Mol Cell Biochem.

[48] Liu J, Chang F, Li F, Fu H, Wang J, Zhang S, Zhao J, Yin D (2015). Palmitate promotes autophagy and apoptosis through ROS-dependent JNK and p38 MAPK. Biochem Biophys Res Commun.

[49] Tang D, Kang R, Livesey KM, Cheh CW, Farkas A, Loughran P, Hoppe G, Bianchi ME, Tracey KJ, Zeh HJ, Lotze MT (2010). Endogenous HMGB1 regulates autophagy. J Cell Biol.

[50] Levine B, Mizushima N, Virgin HW (2011). Autophagy in immunity and inflammation. Nature.

[51] Zhang Q, Kang R, Zeh HJ, Lotze MT, Tang D (2013). DAMPs and autophagy: cellular adaptation to injury and unscheduled cell death. Autophagy.

[52] Oberstein A, Jeffrey PD, Shi Y (2007). Crystal structure of the Bcl-XL-Beclin 1 peptide complex: Beclin 1 is a novel BH3-only protein. J Biol Chem.

[53] Shtivelman E, Hensing T, Simon GR, Dennis PA, Otterson GA, Bueno R, Salgia R (2014). Molecular pathways and therapeutic targets in lung cancer. Oncotarget.

[54] Rivoltini L, Chiodoni C, Squarcina P, Tortoreto M, Villa A, Vergani B, Bürdek M, Botti L, Arioli I, Cova A, Mauri G, Vergani E, Bianchi B (2016). TNF-related apoptosis-inducing ligand (TRAIL)-armed exosomes deliver pro-apoptotic signals to tumor site. Clin Cancer Res.

[55] Zha H, Aime-Sempe C, Sato T, Reed JC (1996). Proapoptotic protein Bax heterodimerizes with Bcl-2 and homodimerizes with Bax via a novel domain (BH3) distinct from BH1 and BH2. J Biol Chem.

[56] Talbert EE, Smuder AJ, Min K, Kwon OS, Powers SK (2013). Calpain and caspase-3 play required roles in immobilization-induced limb muscle atrophy. J Appl Physiol.

[57] Wang X, Song H, Yu Q, Liu Q, Wang L, Liu Z, Yu Z (2015). Ad-p53 enhances the sensitivity of triple-negative breast cancer MDA-MB-468 cells to the EGFR inhibitor gefitinib. Oncol Rep.

[58] Wiley SR, Schooley K, Smolak PJ, Din WS, Huang CP, Nicholl JK, Sutherland GR, Smith TD, Rauch C, Smith CA, Goodwin RG (1995). Identification and characterization of a new member of the TNF family that induces apoptosis. Immunity.

[59] Mahalingam D, Szegezdi E, Keane M, de Jong S, Samali A (2009). TRAIL receptor signalling and modulation: are we on the right TRAIL?. Cancer Treat Rev.

[60] Sheikh MS, Burns TF, Huang Y, Wu GS, Amundson S, Brooks KS, Fornace AJ, el-Deiry WS (1998). p53-dependent and -independent regulation of the death receptor KILLER/DR5 gene expression in response to genotoxic stress and tumor necrosis factor alpha. Cancer Res.

[61] Liu X, Yue P, Khuri FR, Sun SY (2004). p53 upregulates death receptor 4 expression through an intronic p53 binding site. Cancer Res.

[62] Chen G, Wang Y, Li M, Xu T, Wang X, Hong B, Niu Y (2014). Curcumol induces HSC-T6 cell death through suppression of Bcl-2: involvement of PI3K and NF-kappaB pathways. Eur J Pharm Sci.

[63] Kim HY, Kim SL, Park YR, Liu YC, Seo SY, Kim SH, Kim IH, Lee SO, Lee ST, Kim SW (2015). Balsalazide potentiates parthenolide-mediated inhibition of nuclear factor-kappaB signaling in HCT116 human colorectal cancer cells. Intest Res.

[64] Lin MT, Chang CC, Chen ST, Chang HL, Su JL, Chau YP, Kuo ML (2004). Cyr61 expression confers resistance to apoptosis in breast cancer MCF-7 cells by a mechanism of NF-kappaB-dependent XIAP up-regulation. J Biol Chem.

[65] Shingu T, Yamada K, Hara N, Moritake K, Osago H, Terashima M, Uemura T, Yamasaki T, Tsuchiya M (2003). Synergistic augmentation of antimicrotubule agent-induced cytotoxicity by a phosphoinositide 3-kinase inhibitor in human malignant glioma cells. Cancer Res.

[66] Franke TF, Hornik CP, Segev L, Shostak GA, Sugimoto C (2003). PI3K/Akt and apoptosis: size matters. Oncogene.

[67] Wang Y, Kellner J, Liu L, Zhou D (2011). Inhibition of p38 mitogen-activated protein kinase promotes *ex vivo* hematopoietic stem cell expansion. Stem Cells Dev.

[68] Awda BJ, Buhr MM (2010). Extracellular signal-regulated kinases (ERKs) pathway and reactive oxygen species regulate tyrosine phosphorylation in capacitating boar spermatozoa. Biol Reprod.

[69] Papa S, Zazzeroni F, Pham CG, Bubici C, Franzoso G (2004). Linking JNK signaling to NF-kappaB: a key to survival. J Cell Sci.

[70] Moreno E, Yan M, Basler K (2002). Evolution of TNF signaling mechanisms: JNK-dependent apoptosis triggered by Eiger, the Drosophila homolog of the TNF superfamily. Curr Biol.

[71] Dhanasekaran DN, Reddy EP (2008). JNK signaling in apoptosis. Oncogene.

[72] Peng X, Gandhi V (2012). ROS-activated anticancer prodrugs: a new strategy for tumor-specific damage. Ther Deliv.

[73] Nishikawa M, Hashida M (2006). Inhibition of tumour metastasis by targeted delivery of antioxidant enzymes. Expert Opin Drug Deliv.

[74] Park SY, Kim YH (2009). Surfactin inhibits immunostimulatory function of macrophages through blocking NK-κB, MAPK and Akt pathway. Int Immunopharmacol.

[75] Yang TC, Lai CC, Shiu SL, Chuang PH, Tzou BC, Lin YY, Tsai FJ, Lin CW (2010). Japanese encephalitis virus down-regulates thioredoxin and induces ROS-mediated ASK1-ERK/p38 MAPK activation in human promonocyte cells. Microbes Infect.

[76] Son Y, Cheong YK, Kim NH, Chung HT, Kang DG, Pae HO (2011). Mitogen-activated protein kinases and reactive oxygen species: how can ROS activate MAPK pathways?. J Signal Transduct.

[77] Ni HM, Bockus A, Wozniak AL, Jones K, Weinman S, Yin XM, Ding W (2011). Dissecting the dynamic turnover of GFP–LC3 in the autolysosome. Autophagy.

[78] Shen MX, Ding JB (2017). Expression levels and roles of EMC-6, Beclin1, and Rab5a in the cervical cancer. Eur Rev Med Pharmacol Sci.

[79] Li W, Xiong L, Shi X, Xiao L, Qi G, Meng C (2016). IKKβ/NFκBp65 activated by interleukin-13 targets the autophagy-related genes LC3B and beclin1 in fibroblasts co-cultured with breast cancer cells. Exp Ther Med.

[80] Amelio I, Melino G, Knight RA (2011). Cell death pathology: cross-talk with autophagy and its clinical implications. Biochem Biophys Res Commun.

[81] González-Polo RA, Boya P, Pauleau AL, Jalil A, Larochette N, Souquère S, Eskelinen EL, Pierron G, Saftig P, Kroemer G (2005). The apoptosis/autophagy paradox: autophagic vacuolization before apoptotic death. J Cell Sci.

[82] Zou Y, Bu H, Guo L, Liu Y, He J, Feng X (2016). Staining with two observational methods for the diagnosis of tuberculous meningitis. Exp Ther Med.

[83] Cruz FF, Antunes MA, Abreu SC, Fujisaki LC, Silva JD, Xisto DG, Maron-Gutierrez T, Ornellas DS, Sá VK, Rocha NN, Capelozzi VL, Morales MM, Rocco PR (2012). Protective effects of bone marrow mononuclear cell therapy on lung and heart in an elastase-induced emphysema model. Resp Physiol Neurobiol.

[84] Guan XJ, Song L, Han FF, Cui ZL, Chen X, Guo XJ, Xu WG (2013). Mesenchymal stem cells protect cigarette smoke-damaged lung and pulmonary function partly via VEGF-VEGF receptors. J Cell Biochem.

