# Supplementary material for: Juglanin inhibits lung cancer by regulation of apoptosis, ROS and autophagy induction
Source: Oncotarget. 2017 Sep 28;8(55):93878–98. doi: 10.18632/oncotarget.21317 (PMC5706842; doi:10.18632/oncotarget.21317)
Supplement: Supplementary file 1 [file oncotarget-08-93878-s001.pdf]

Juglanin inhibits lung cancer by regulation of apoptosis, ROS and autophagy induction

SUPPLEMENTARY MATERIALS

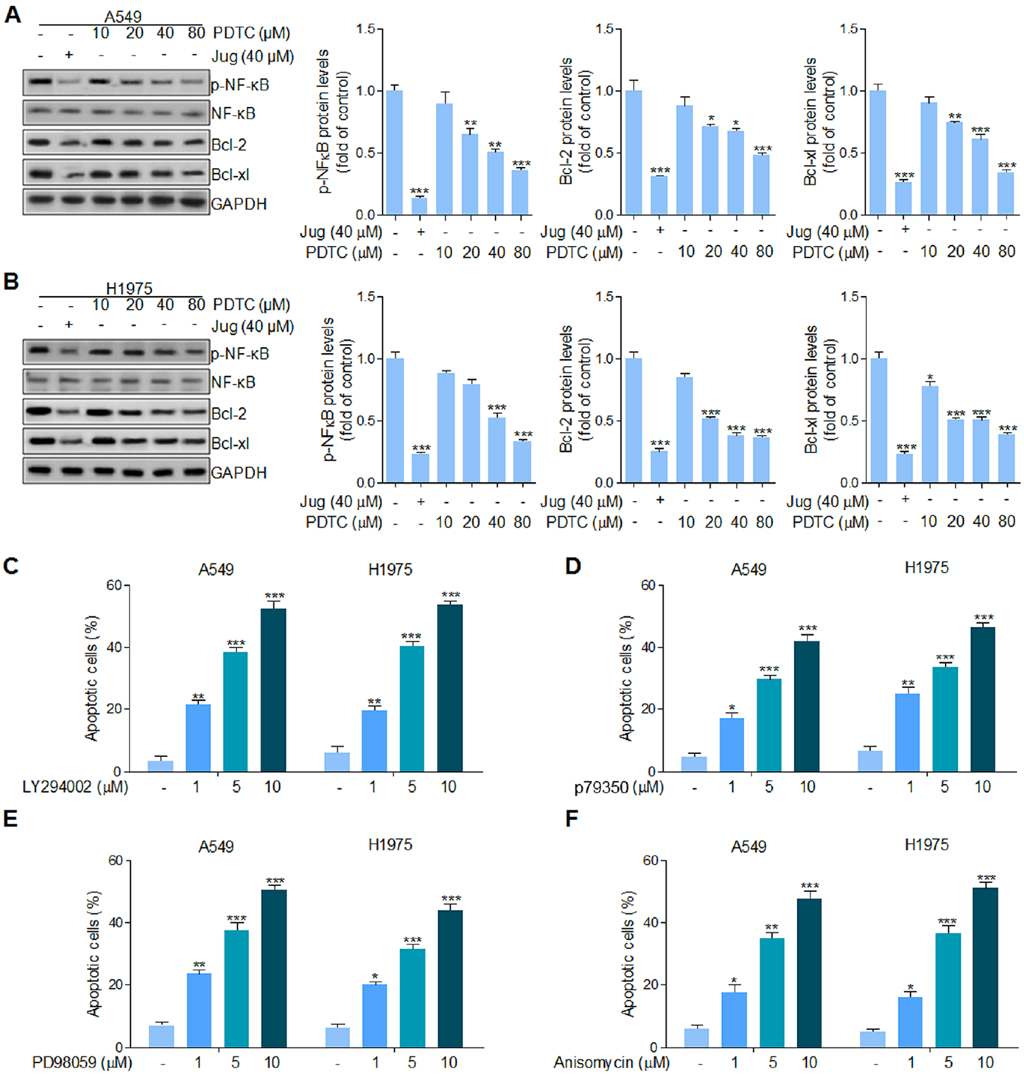

Supplementary Figure 1: NF-κB and MAPKs signaling pathways were involved in juglanin-suppressed lung cancer cells. **(A)** A549 and **(B)** HCC827 cells were treated with different concentrations of PDTC, NF-κB inhibitor, in the presence or absence of 40 μM juglanin for 24 h. Then, immunoblotting analysis was conducted to assess NF-κB phosphorylation and Bcl-2, as well as Bcl-xl protein levels. Left, the representative images of western blotting analysis were displayed. Right, the quantification following immunoblotting analysis was shown. **(C)** A549 and HCC827 cells were treated with PI3K/AKT inhibitor LY294002 under different concentrations for 12 h, and the flow cytometry was used to calculate the apoptotic cells. **(D)** A549 and HCC827 cells were treated with p38 activator p79350 with different concentrations for 12 h, followed by flow cytometry to assess the apoptotic cells. **(E)** A549 and HCC827 cells were administrated with ERK1/2 inhibitor PD98059 at different concentrations for 12 h. Next, the apoptotic cells was determined by flow cytometry. **(F)** A549 and HCC827 cells were treated with JNK activator Anisomycin at different concentrations for 12 h, and then the percentage of apoptotic cells was measured. The data are presented as mean ± S.E.M. of three separate experiments performed in duplicate. ** $P < 0.01$ and *** $P < 0.001$ compared to Control group (Con) without any treatment.

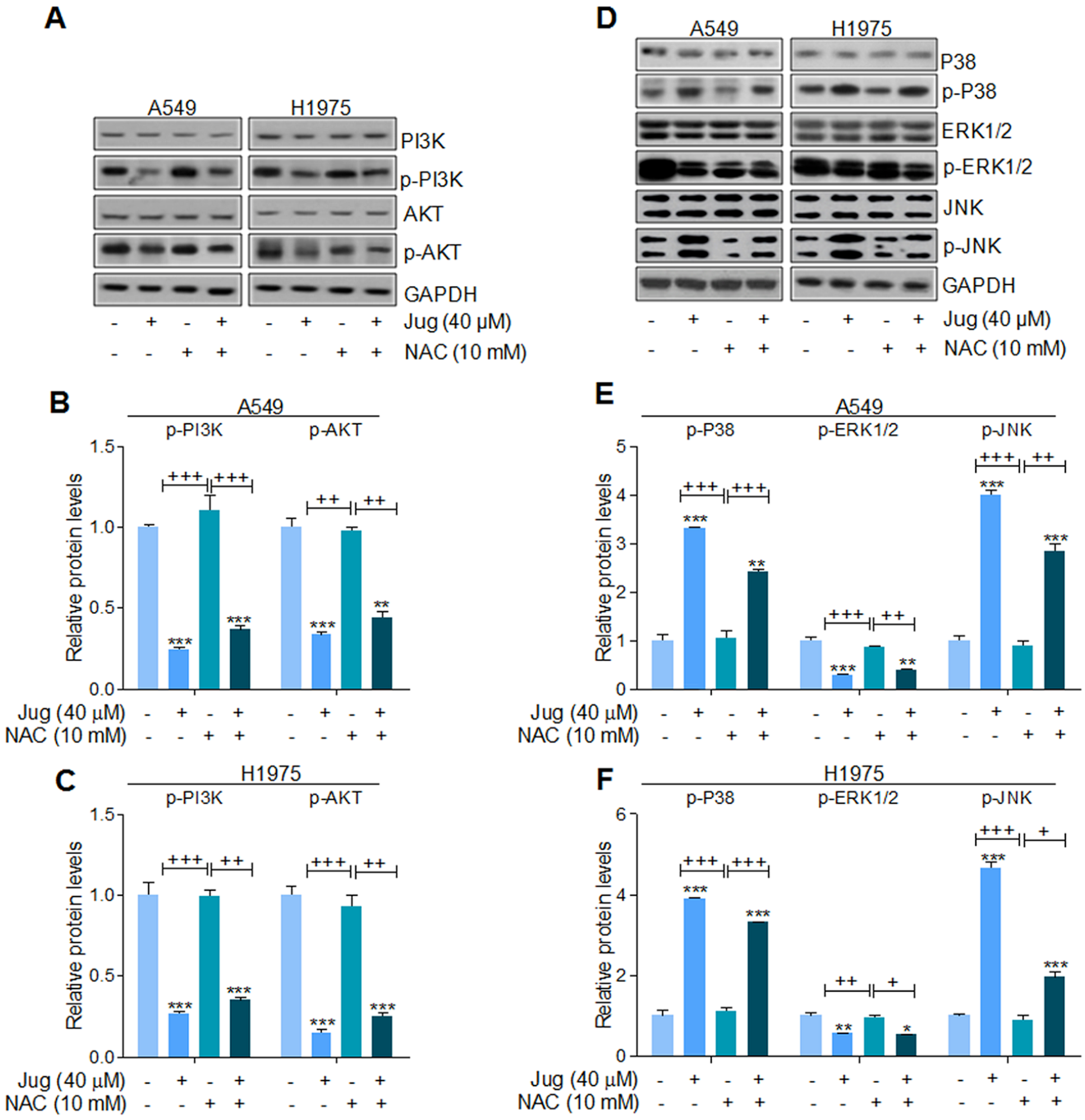

Supplementary Figure 2: ROS-regulated PI3K/AKT and MAPKs signaling pathways were involved in Juglanin-treated lung cancer. **(A)** A549 and HCC827 cells were treated with juglanin and NAC, ROS inhibitor, under various conditions for 24 h, followed by western blot analysis. The representative images were shown. The quantification of p-PI3K and p-AKT in **(B)** A549 and **(C)** HCC827 cells was exhibited. **(D)** A549 and HCC827 cells were cultured with or without juglanin and NAC for 24 h first. Then, the cells were harvested for western blotting analysis. The quantification of p-p38, p-ERK1/2 and p-JNK in **(E)** A549 and **(F)** HCC827 cells was displayed. The data are presented as mean ± S.E.M. of three separate experiments performed in duplicate. ** $P < 0.01$ and *** $P < 0.001$ compared to Control group (Con) without any treatment. $^{+}$ $P < 0.05$, $^{++}$ $P < 0.01$ and $^{+++}$ $P < 0.001$ were considered with significant difference.
